# Novel long non-coding RNAs are specific diagnostic and prognostic markers for prostate cancer

**DOI:** 10.18632/oncotarget.2879

**Published:** 2015-02-17

**Authors:** René Böttcher, A. Marije Hoogland, Natasja Dits, Esther I. Verhoef, Charlotte Kweldam, Piotr Waranecki, Chris H. Bangma, Geert J.L.H. van Leenders, Guido Jenster

**Affiliations:** ^1^ Dept. of Urology, Erasmus MC, Rotterdam, The Netherlands; ^2^ Dept. of Pathology, Erasmus MC, Rotterdam, The Netherlands; ^3^ Dept. of Bioinformatics, Technical University of Applied Sciences Wildau, Wildau, Germany

**Keywords:** Long non-coding RNA, prostate cancer, *in situ* hybridization, exon array, biomarkers

## Abstract

Current prostate cancer (PCa) biomarkers such as PSA are not optimal in distinguishing cancer from benign prostate diseases and predicting disease outcome. To discover additional biomarkers, we investigated PCa-specific expression of novel unannotated transcripts. Using the unique probe design of Affymetrix Human Exon Arrays, we identified 334 candidates (EPCATs), of which 15 were validated by RT-PCR. Combined into a diagnostic panel, 11 EPCATs classified 80% of PCa samples correctly, while maintaining 100% specificity. High specificity was confirmed by *in situ* hybridization for EPCAT4R966 and EPCAT2F176 (SChLAP1) on extensive tissue microarrays. Besides being diagnostic, EPCAT2F176 and EPCAT4R966 showed significant association with pT-stage and were present in PIN lesions. We also found EPCAT2F176 and EPCAT2R709 to be associated with development of metastases and PCa-related death, and EPCAT2F176 to be enriched in lymph node metastases. Functional significance of expression of 9 EPCATs was investigated by siRNA transfection, revealing that knockdown of 5 different EPCATs impaired growth of LNCaP and 22RV1 PCa cells. Only the minority of EPCATs appear to be controlled by androgen receptor or ERG. Although the underlying transcriptional regulation is not fully understood, the novel PCa-associated transcripts are new diagnostic and prognostic markers with functional relevance to prostate cancer growth.

## INTRODUCTION

Despite continuous research efforts over the past decades, prostate cancer (PCa) remains one of the leading causes of male cancer deaths, with an estimated 70,100 deaths in Europe in 2014. Incidence rates are highest in countries of the western hemisphere including Europe, North America and Oceania, which can be partly explained by the widely applied blood test for prostate specific antigen (PSA) [1, 2]. Although the serum PSA level offers high sensitivity for PCa detection, its specificity is limited as PSA levels can also be elevated in benign prostate diseases such as benign prostate hyperplasia (BPH) and prostatitis. Thus, the most important drawback of PSA screening is a high number of false positives leading to unnecessary biopsies and overtreatment of patients due to a lack of prognostic markers. Up to date this remains a challenge and additional prognostic factors, such as disease associated genes, are needed [3].

Earlier studies discovered several other PCa-associated genes, among them two long non-coding RNAs (lncRNAs) that show disease-associated overexpression, PCGEM1 and PCA3 (DD3) [4, 5]. The latter has since been extensively studied as diagnostic urine marker for PCa, offering better performance for detecting PCa when compared to PSA [6]. With the introduction of high throughput technologies, such as tiling arrays and next generation sequencing, several other PCa-associated lncRNAs such as PRNCR1, PCAT1, PCAT18, PCAT29 and SChLAP1 were identified [7–14].

LncRNAs have been associated with several functions, including epigenetic regulation of gene expression by acting as regulatory factors in *cis*, as well as in *trans* by involvement in chromatin remodeling [15–18]. Additionally, direct binding to active androgen receptor (AR) and recruitment of additional factors for AR-mediated gene expression has been reported [19]. However, a recent study found contradicting evidence for these findings and thus further research is required to clarify lncRNA involvement in AR activity [20]. Still, many functional relationships of lncRNAs as well as their tissue-specific regulation remain unclear. Currently, lncRNAs are gaining more interest as potential biomarkers for various malignant diseases, due to their highly tissue-specific expression profiles [17, 21].

In this study, we set out to discover novel PCa-specific lncRNAs based on Affymetrix Human Exon Arrays by adapting a cancer outlier profile analysis (COPA, [22]). Our approach made use of the unique design of these arrays, which include probes against predicted sequences (‘full’) next to probes targeting known sequences (‘core’ and ‘extended’). This type of microarray has recently been successfully adapted for lncRNA profiling, showing the general potential of the platform in lncRNA studies [11]. To increase reliability of our results, we combined three Affymetrix Human Exon Array datasets and searched for reoccurring outlier patterns indicating novel transcripts. We then used RNA-sequencing (RNA-seq) data to refine our transcript definitions and subsequently validated them via RT-PCR. Computational evaluation of the validated transcripts confirmed absence of protein coding potential, suggesting that these transcripts are indeed lncRNAs. Two transcripts were chosen for staining of tissue microarrays using *in situ* hybridization and successfully discriminated PCa from normal adjacent prostate (NAP) and benign prostate tissue.

## RESULTS

### 334 candidate PCa-associated transcripts were identified

**Figure 1:**
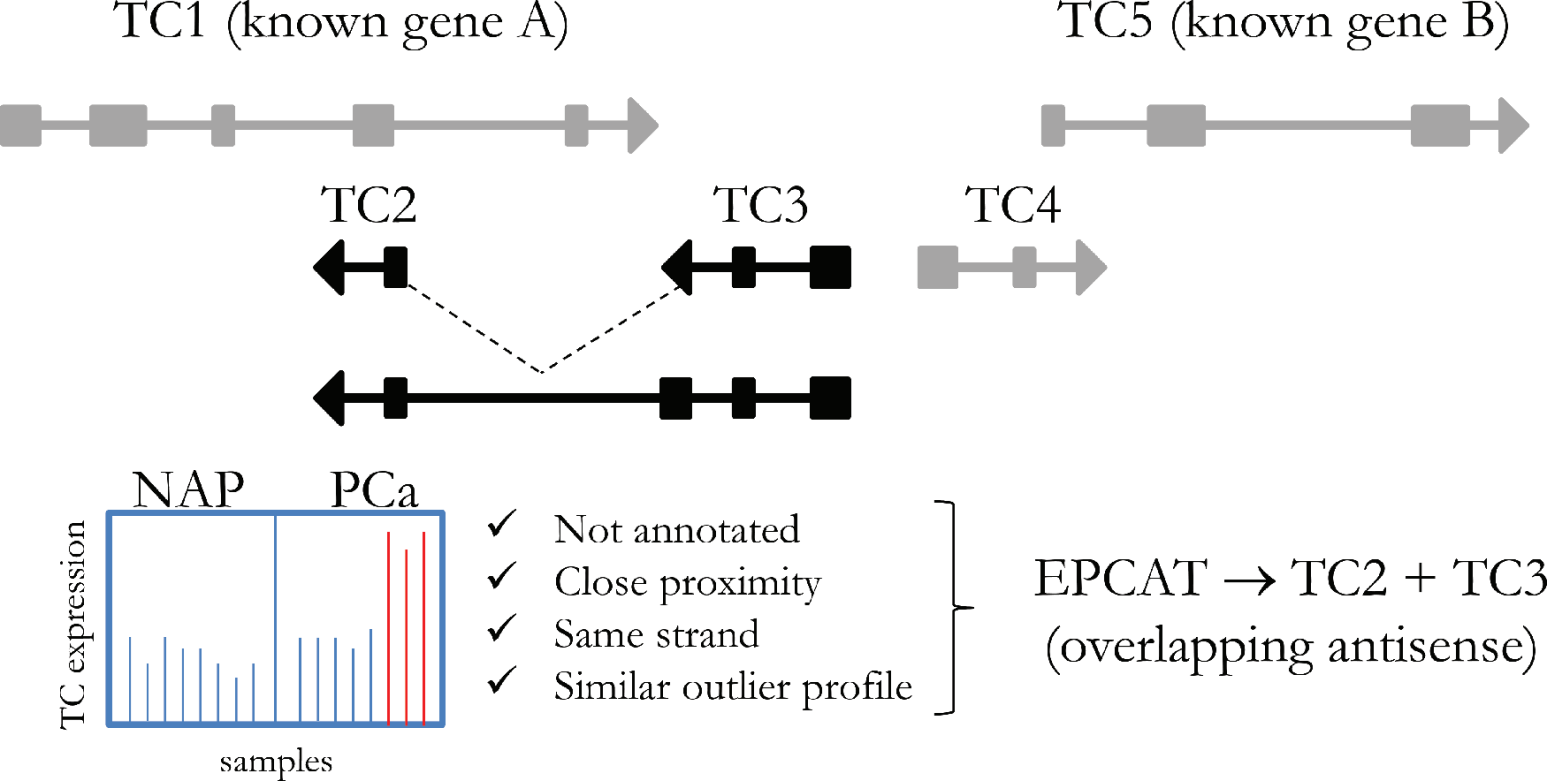
Principle steps of EPCAT identification Affymetrix transcript clusters that had no annotation assigned were grouped into one locus if they were located on the same strand in close proximity (< 250 kb) and showed a similar PCa-specific outlier profile (transcript clusters TC2 and TC3). Transcript clusters that did not meet these criteria were not included in the particular EPCAT.

**Figure 2:**
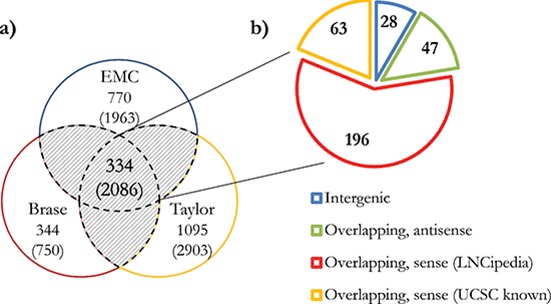
Total number and classification of EPCATs **(a)** Total number of EPCATs identified by each individual dataset as well as a combination of at least two datasets (shaded area, 334 EPCATs). **(b)** Classification of these 334 EPCATs based on their relative position to LNCipedia [23] genes. UCSC known gene annotations were selected if no overlap with LNCipedia was found. Overlaps include cases in which an EPCAT overlaps and extends the 5′ or 3′ ends of known genes or resides in an intron.

Novel transcript candidates were identified by searching for unannotated Affymetrix Human Exon Array transcript clusters (TCs) that showed a PCa-specific outlier profile using a COPA transformation [22]. After removing all TCs targeting known genes, we discarded TCs with fewer than 5% outliers in cancerous samples and with outliers in control groups. All remaining TCs were then grouped into ‘EPCATs’ (Erasmus MC PCa-associated transcripts) based on proximity, strand and similarity in expression (see Figure 1). EPCAT names were assigned to directly indicate genomic location and are based on chromosome, strand and a unique identifier. For instance, EPCAT2F176 (SChLAP1) is located on the forward strand of chromosome 2. EPCATs had to be present in at least two datasets to be considered for further analysis. Differences between datasets (i.e. missing parts in one or the other) were resolved by a union of all TCs involved in a particular EPCAT to maximize its size. Our meta-analysis of three available Exon Array datasets resulted in 334 EPCATs comprising 2086 TCs that exhibited a prostate cancer-specific expression profile (see Supplementary Tables 1–2). We observed that combining several datasets severely reduced the number of EPCATs identified by one dataset alone, suggesting a reduction in false positives in doing so (see Figure 2a). Next, we classified the identified EPCATs based on their genomic origin with regard to UCSC known genes, and observed that 75 EPCATs were being classified as intergenic or antisense transcripts. The majority of EPCATs (259) overlapped/extended either 5′ or 3′ ends or was located in intronic regions of genes known to LNCipedia [23] or UCSC (see Figure 2b).

**Figure 3:**
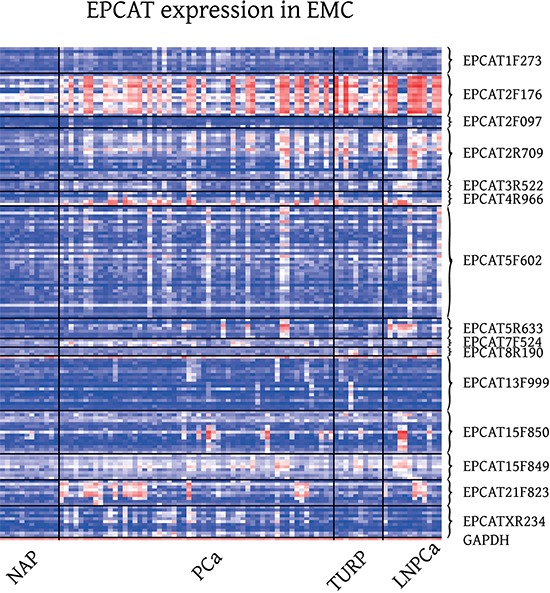
Expression of 15 RT-PCR validated EPCATs in EMC Exon Array samples EMC (GSE41408, [27]), comprised localized prostate cancer obtained via radical prostatectomy (PCa), transurethral resection of the prostate (TURP), lymph node metastasis (LNPCa) and normal adjacent prostate (NAP) tissue.

Visual inspection of these results confirmed that similar PCa-specific expression patterns occurred in all three datasets with TCs grouped into one EPCAT following the same PCa-specific outlier profile (see Figure 3 and Supplementary Figures 1–6 for a subset of 15 EPCATs that were subsequently PCR-validated). We also inspected EPCAT expression in other publicly available datasets comprising samples from lung, brain, breast, colorectal and gastric cancer tissue as well as several normal tissues. For most of the EPCATs, expression was very low in virtually all samples, indicating a PCa-specific expression of these transcripts similar to other previously reported lncRNAs ([12, 24], see Supplementary Figures 3–6). However, some EPCATs such as EPCAT5R633 and EPCATXR234 were detected in multiple lung, colorectal and breast tumors and appear deregulated in different cancer types. To gain insight into their transcriptional regulation, we tested whether any EPCATs are androgen regulated by incorporating a publicly available dataset of R1881 treated LNCaP cells. We observed that out of 301 EPCATs expressed in LNCaP 31 were significantly associated with androgen treatment and showed more than 50% increase or decrease in expression (*p* < 0.05; 13 up-, 18 downregulated, see Supplementary Figure 7). In addition, we tested for coexpression with known outlier genes ERG and ETV1 [22] by Spearman's correlation coefficient, and found that 17 EPCATs showed significant correlation with ERG (Spearman's *p* ≥ 0.5 and *p* < 0.05, see Supplementary Figure 8), while no significant coexpression with ETV1 was observed. Public ChIP-seq data [25] targetting AR and ERG was used as second source of evidence for AR and ERG regulation. We found that 15 of the 33 differentially expressed EPCATs (including 50 kb flanks) had overlapping AR peaks, whereas ERG peaks were found for 4 of the 17 coexpressed EPCATs (see methods).

**Figure 4:**
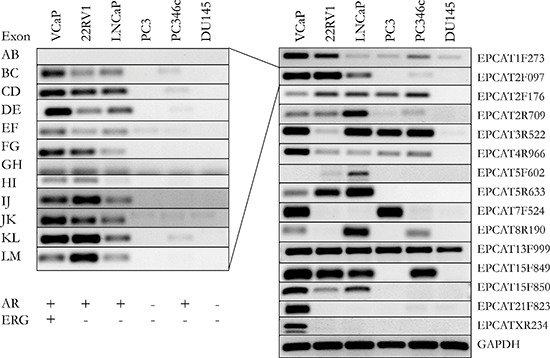
Validation of 15 EPCATs in 6 prostate cancer cell lines Intron-spanning primers were designed for each EPCAT. Exons of one transcript followed similar expression patterns (left side). Only the most representative and optimal primer set for an EPCAT is shown in the right panel. These primers were also used to design Taqman probes (see Supplementary Tables 9–10). AR and TMPRSS2-ERG status for each cell line are indicated as present (+) or absent (−).

To gather more evidence for the existence of our transcript candidates, we performed a reference guided assembly of RNA-seq data obtained from 18 patients with localized PCa as well as 5 samples from lymph node metastases. We used Cufflinks [26] to predict intron-exon boundaries in the genomic regions of the EPCATs while masking known annotated genes, which resulted in 222 predicted transcripts. We chose 20 well defined candidates that showed high expression and added additional candidate exons after manual evaluation of several genomic loci. We also included EPCAT8R190, which was initially filtered out due to its presence in only one dataset (EMC), but was subsequently discovered as a candidate due to its high expression in castration resistant prostate cancer (CRPC). We were able to design working RT-PCR primers for 15 out of these 21 candidates and validated their expression in 6 prostate cancer cell lines (see Figure 4 and Supplementary Table 3). The primers were designed intron spanning, allowing us to PCR from exon to exon, and validated exons were Sanger sequenced. Individual exons of an EPCAT showed the same expression pattern throughout our cell line panel, whereas expression patterns differed between different EPCATs, indicating independent expression and regulation. To obtain full length sequences, a λgt11 library containing cDNA from the LNCaP cell line was used (see Materials and Methods).

### EPCATs can serve as diagnostic markers in patient tissues

TaqMan RT-PCR was used to quantify expression of the 15 EPCATs in two separate patient cohorts, however, only 11 EPCATs had working TaqMan probes and were subsequently quantified. The first cohort comprised a subset of patients also present in the EMC Exon Array dataset and allowed comparison between qRT-PCR and Exon Arrays for the EPCATs. Therefore, we treated this cohort as a training set and used the second, independent cohort as validation set. Comparing expression measurements of qRT-PCR with the averaged expression values of all TCs of an EPCAT yielded varying concordance between both techniques (average *R^2^* = 0.58, see Supplementary Figure 9). These results indicated that not all EPCATs were sufficiently represented by Affymetrix TCs and that RNA-seq data is essential for defining gene structures. Next, a receiver operator characteristic (ROC) was created using the test cohort to maximize area under curve (AUC) by weighing each EPCAT in the diagnostic panel. When applying the same panel to the validation cohort, an AUC of 0.87 confirmed high specificity and sensitivity for PCa diagnosis (see Supplementary Figure 10).

### Two lncRNAs in 2q31.3 are associated with prostate cancer progression

To evaluate possible prognostic value of the 15 validated EPCATs from our EMC Exon Array dataset, we characterized their expression profiles in 54 patients with clinical follow-up (see [27] for further information). We performed a retrospective analysis for prediction of prostate cancer-related death (PCaD), development of clinical metastases (PCaMets) after radical prostatectomy (RP) as well as biochemical recurrence (BCR) defined by PSA progression after RP. Samples were clustered into two groups using Partition Around Medoids (PAM) and significant association with clinical endpoints was tested using a bootstrapping analysis and label permutation to calculate *p*-values (see methods). Using FDR correction, we observed that EPCAT2R709 and EPCAT2F176 (SChLAP1) showed significant association with PCaMets and PCaD. To evaluate whether any EPCAT could discriminate poor clinical outcome, we used a Kaplan-Meier analysis for the same clinical endpoints. Again, EPCAT2F176 and EPCAT2R709 showed a significant association with PCaMets and PCaD (see Supplementary Figures 11–14 and Supplementary Tables 4a–4d). Interestingly, both EPCAT loci are located in chromosome 2q31.3, with EPCAT2R709 being found on the antisense strand, approximately 120 kb upstream of the first exon of EPCAT2F176. Additionally, both EPCATs show similar expression profiles (Spearman's *p* = 0.79 for all samples analyzed via qRT-PCR, *p* = 0.93 for EMC Exon Arrays).

### Evaluation of coding potential and conservation

We evaluated if any of the 15 PCR-validated EPCATs exhibits protein coding potential using two approaches: iSeeRNA and PhyloCSF [28, 29]. iSeeRNA classified all processed EPCATs as non-coding, however, EPCAT13F999 did not pass minimum length requirements (200 bp). We used all known coding RefSeq genes (36,818) as positive control, of which 34,476 (93.64%) were classified as protein coding and 2342 (6.36%) as non-coding. For PhyloCSF, known coding genes GAPDH and ERG were used as positive controls. Both genes were assigned high positive scores by PhyloCSF, as compared to negative scores for all EPCATs indicating no coding potential (see Supplementary Figure 15). Sequence conservation of the EPCATs was evaluated using per-base conservation scores from UCSC (PhyloP) for several genome panels. 1000 randomly picked coding genes in the UCSC RefSeq table as well as 1000 repeat regions served as controls. The results illustrate that EPCAT sequences are overall less conserved than protein coding sequences, while being more conserved than most repeat regions, which is concordant with previous findings ([17, 24], see Supplementary Figure 16).

### *In situ* hybridization revealed diagnostic power and prognostic value

**Figure 5:**
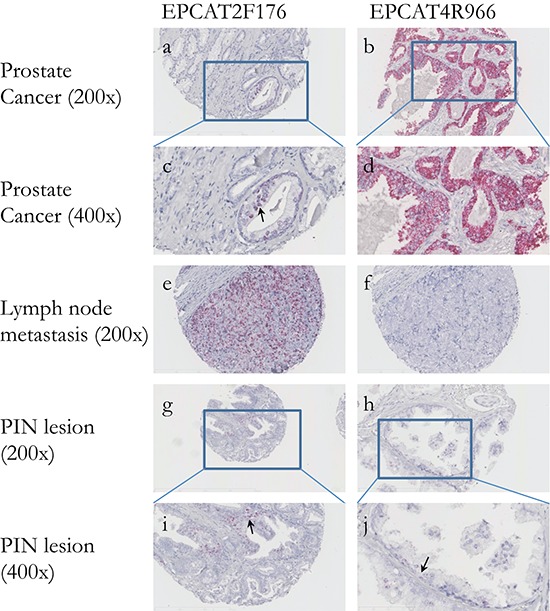
*In situ* hybridization of two EPCATs in prostate cancer tissues **(a–d)** Both EPCAT2F176 as well as EPCAT4R966 show highly specific expression in PCa cells, whereas surrounding stromal tissue scored negative. **(e–f)** Lymph node metastases also scored positive for both EPCATs and complementary expression could be observed when comparing the same tissue cores, highlighting their added diagnostic potential. **(g–j)** PIN lesions were also found positive, indicating EPCAT expression as an early event in cancer development.

**Figure 6:**
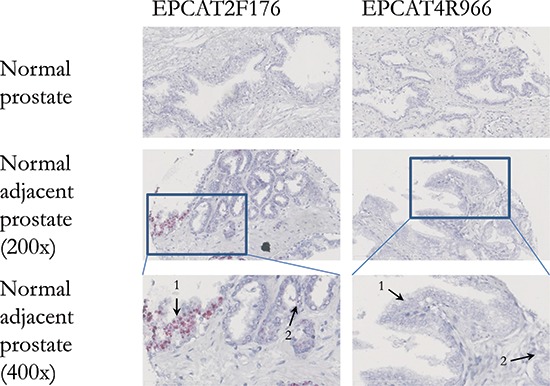
*In situ* hybridization of two EPCATs in normal prostate tissues Both EPCAT2F176 as well as EPCAT4R966 showed no expression in normal prostate tissue obtained via radical cystoprostatectomy. However, normal cells (1) adjacent to prostate cancer (2) were found positive for both EPCATs.

To investigate whether EPCATs can serve as potential pathological tissue markers markers and specifically distinguish cancerous from normal prostate tissues, we stained tissue microarrays (TMAs) for presence of the two EPCATs showing highest expression among our 11 qRT-PCR quantified transcripts (EPCAT2F176/SChLAP1 and EPCAT4R966). Due to their non-coding nature, we used *in situ* hybridization (ISH) to directly target the RNA molecules. All four TMAs comprised a total of 418 PCa samples from RPs, 120 transurethral resections of the prostate (TURP, 65 hormone refractory, 55 hormone sensitive), 119 lymph node metastasis (LNPCa) and 113 normal adjacent prostate samples (NAP), as well as normal prostate obtained via 81 TURPs, 5 total pelvic exenterations (TE) and 48 radical cystoprostatectomies (RCP). Normal tissue samples from kidney, liver, placenta as well as a sample containing urothelial cell carcinoma served as control (see Supplementary Tables 5a–5b). After TMA scoring, we observed that all 4 control tissues on TMA 1 and 2 were indeed negative (score = 0) for both EPCATs, which showed PCa-specific expression as expected from our previous findings (see Figure 5a–5j and Supplementary Table 6). Moreover, we found significant association with pathological stage, whereas other clinical parameters (Gleason score, surgical margins, pre-treatment PSA) were not significantly associated (see Supplementary Tables 7a–7d). Normal prostate samples of patients without prostate cancer showed complete absence of EPCAT expression (see Supplementary Figure 16), whereas 12 NAP samples (10.62%) exhibited higher expression levels compared to samples from normal prostate (Figure 6). In a ROC analysis, both EPCATs showed high specificity and limited sensitivity in distinguishing cancerous samples when used individually (28.61% PCa samples positive, AUC = 0.66 for EPCAT2F176/SChLAP1 and 28.01% PCa samples positive, AUC = 0.65 for EPCAT4R966). Combining both EPCATs, we were able to correctly classify 39.4% of the cancer samples in our cohort while maintaining a specificity of 100% (AUC = 0.71).

Using ISH also allowed us to study subcellular localization of the EPCATs, revealing that both transcripts are present in the cytoplasm as well as the nucleus, with EPCAT2F176 showing a tendency to be more nuclear than cytoplasmic, consistent with previous findings [12]. Furthermore, we also identified several prostate intraepithelial neoplasia (PIN) lesions that showed positive staining for the EPCATs (7/21 lesions for EPCAT2F176 (33.3%), 1/21 lesion for EPCAT4R966 (4.8%), see Figure 6g–6j).

We used our third TMA comprising 119 samples to evaluate EPCAT expression in lymph nodes of patients undergoing a lymph node exploration in addition to RP. We found that out of 73 samples containing tumor tissue, 46 were positive for EPCAT2F176 (63.0%), representing a significant increase in number of positive samples compared to localized PCa (*p* = 0.0404, Fisher's exact test). For EPCAT4R966, tumor was present in 71 of the sliced cores, of which 16 were stained positive (22.5%; *p* = 0.3866, Fisher's exact test). Furthermore, all tumor free samples were found to be negative.

As for our fourth TMA comprising hormone refractory and hormone sensitive patient samples, we did not observe a significant correlation of hormonal status with any EPCAT nor a combination of both. EPCAT2F176 was found positive in 61 out of 109 TURP samples containing tumor tissue (55.9%), whereas 41 out of 103 tumor containing samples were positive for EPCAT4R966 (39.8%, see Supplementary Table 6).

### Knock-down of EPCATs impedes growth of prostate cancer cells

**Figure 7:**
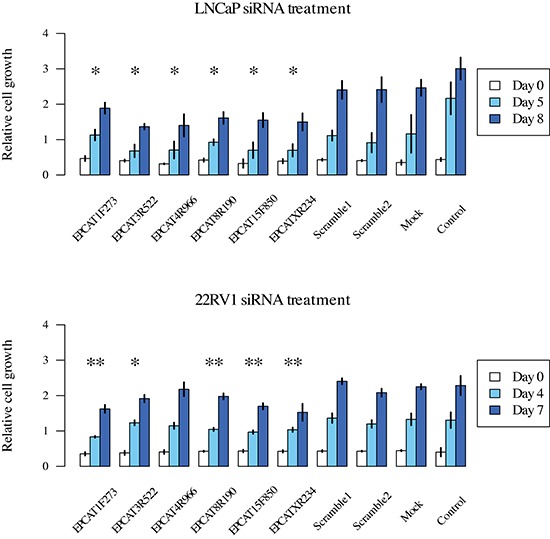
Cell viability measured by MTT assay after treatment of LNCaP and 22RV1 cells All measurements were performed in triplicates and a t-test was used to determine significant differences (*p* < 0.05) between treatment and scrambled control RNA. *denotes a significant difference at day 7/8, **at both day 5 and 8/day 4 and 7 for LNCaP and 22RV1, respectively. Experiments were performed twice and representative results are displayed.

To investigate their functional impact on PCa growth, we performed siRNA-directed knockdown of 9 PCR-validated EPCATs (EPCAT1F273, EPCAT2F176, EPCAT2R709, EPCAT3R522, EPCAT4R966, EPCAT5R633, EPCAT8R190, EPCAT15F850, EPCATXR234) in LNCaP and 22RV1 cells. Cell viability was assessed by MTT-assay, and transfections with two scrambled RNAs were used to evaluate unspecific treatment effects of siRNA transfection. We observed significant reductions in cell viability for 6 of these 9 EPCATs (EPCAT1F273, EPCAT3R522, EPCAT4R966, EPCAT8R190, EPCAT15F850, EPCATXR234), 5 of which were showing consistent effects in both LNCaP and 22RV1 (see Figure 7 and Supplementary Figure 17).

## DISCUSSION

We successfully set out to identify novel transcripts with PCa-specific expression profiles using unannotated transcript clusters of Affymetrix Human Exon Arrays. The large number of transcript candidates identified shows that we do not yet have a full overview of all the transcribed genomic regions. With efforts such as ENCODE and GENCODE, it has become clear that the number of protein coding genes is reaching a plateau of about 21,000 [30]. On the contrary, the number of non-coding transcripts is increasing rapidly, as particularly deep RNA-sequencing of many normal and diseased tissues reveals a wealth of novel small and long transcripts. Our 334 EPCATs add to this pool of newly identified RNAs. 10 EPCATs were also identified by Prensner *et al*., while 196 EPCATs, of which 9 validated transcripts, overlapped with the 32,183 human transcripts present in the LNCipedia [23] database.

In previous studies, several lncRNAs have been associated with PCa development and progression, emphasizing their role as potential markers and therapy targets in cancers [17]. Various mechanisms of lncRNA dependent activation and repression of expression have been reported in PCa, among them are post-transcriptional regulation of BRCA2 by PCAT-1 [31], post-translational regulation of SNF5 protein by SChLAP1 binding [12] as well as mediation of enhancer-promoter looping by interaction with AR (PCGEM1 and PRNCR1, [19]), which is currently disputed and requires further research for clarification [20]. Other described mechanisms include regulation of alternative splicing by MALAT1 and silencing of antisense genes by CDKN2B-AS1/ANRIL [32]. Furthermore, PCAT29 (EPCAT15F849) has been recently suggested as tumor suppressor in PCa, although its mechanism of action is still unclear [13].

Despite these promising findings, the value of the newly identified lncRNAs in PCa prognostic profiles has not yet been established. To address the need for novel prognostic markers, we investigated whether EPCAT expression on three Affymetrix Exon Array cohorts is related to poor prognostic outcome and found that at least two transcripts (EPCAT2F176/SChLAP1 and EPCAT2R709) are associated with development of metastasis and PCa-related death. EPCAT2R709 is located approximately 120 kb upstream in antisense direction to EPCAT2F176, making the genomic region on chromosome 2q31.3 a highly interesting target for further studies. Using the RNAscope ISH technology, we independently validated the diagnostic accuracy and power to predict pathological stage of EPCAT2F176 and EPCAT4R966. The association of EPCAT2F176 with development of metastasis and PCa-related death was not confirmed using the TMA, which could be due to differences in sample cohorts and detection technologies. Nevertheless, we did observe a significant increase in number of positive LNPCa samples compared to localized PCa for EPCAT2F176, which could indicate an involvement in formation of metastasis and supports our earlier results.

Both EPCAT2F176 and EPCAT4R966 were found expressed in some PIN lesions by ISH, suggesting that their expression might be an early event in PCa development. Moreover, both transcripts were expressed in approximately 10% of NAP tissue samples, whereas normal prostate controls were completely negative, suggesting that normal adjacent tissue might differ from truly normal tissue as previously reported [33–35]. Therefore, lncRNA biomarkers such as our EPCATs enable a morphology-independent, molecular-based identification of potentially malignant prostate tissue. Taken together, these findings highlight the high specificity of EPCAT expression and pose questions as to how these lncRNAs are regulated and why they are expressed in subsets of patients only.

We chose three transcription factors with known involvement in PCa to investigate EPCAT regulation, namely AR, ERG and ETV1. Using public Affymetrix Exon Array [36] and ChIP-seq data [25] we found evidence for 4 ERG and 15 AR regulated EPCATs, of which 3 had been PCR-validated. Since the majority of EPCATs does not appear to be AR or ERG regulated, other regulatory mechanisms such as DNA methylation, chromatin restructuring or combinations of transcription factors could play a role. Thus, whether an interplay between these factors will explain the outlier PCa-specific expression of EPCATs is a new and challenging field of research.

In addition to their reported diagnostic and prognostic potential, siRNA-directed knockdown in combination with an MTT-assay revealed that 6 EPCATs (EPCAT1F273, EPCAT3R522, EPCAT4R966, EPCAT8R190, EPCAT15F850, EPCATXR234) are involved in PCa cell viability and growth. Like the recently identified PCAT1, SChLAP1 and PCAT29, the expression of some of the novel EPCATs is functionally relevant and therefore, cancer-associated lncRNAs should not entirely be seen as transcriptional noise due to aberrant regulation.

Despite unknown regulation of most EPCATs, they offer high specificity in discriminating malignant disease from benign prostate tissues. With the exemplary lncRNA PCA3 being used as clinical diagnostic marker in a urine-based test [6], one can envision that a combination of EPCATs can supplement PCA3 and TMPRSS2-ERG based diagnostic panels. If EPCATs are present in urine, such an assay might help to improve specificity of diagnosis of current markers and reduce the number of unnecessary prostate biopsies.

In conclusion, we present evidence for the existence of novel prostate cancer-specific transcripts that demonstrate diagnostic and prognostic value and might serve important roles in tumor development and progression. A subset of EPCATs is Androgen Receptor or ERG regulated, but for most novel transcripts their unique transcriptional regulation in cancer is still not fully resolved and poses a new challenging research question.

## METHODS

### Public exon array datasets

We used three independent publicly available datasets of Affymetrix Human Exon Arrays to discover novel prostate cancer-associated transcripts; referred to as ‘Taylor’ (GSE21034, [37]) and ‘Brase’ (GSE29079, [38]) and ‘EMC’. ‘EMC’ contains 48 previously published prostate cancer samples (GSE41408, [27]) as well as additional cancerous and control samples, accessible via GEO accession number GSE59745. The datasets comprised samples from normal adjacent prostate (NAP), localized prostate cancer obtained via radical prostatectomy (PCa) and transurethral resection of the prostate (TURP, EMC only), as well as metastasis in lymph node (LNPCa, EMC and Taylor) and other tissues (MetPCa, Taylor only). Public datasets of other tissues were used for validation of PCa-specific expression and contained samples of lung cancer (GSE12236, [39]), gastric cancer (GSE13195), brain cancer (GSE9385, [40]) as well as breast, colorectal and lung cancer tissue (GSE16534, [41, 42]). Androgen regulation of novel transcripts was investigated using a public dataset of LNCaP cells grown in androgen depleted medium or in presence of 10 nM R1881 (GSE32875, [36]).

### Patient samples used for gene expression microarray, qRT-PCR and tissue microarray analysis

We used normal and tumor samples of patients from the frozen tissue bank of the Erasmus Medical Center (Rotterdam, the Netherlands, obtained between 1984 and 2001). Further information concerning these patient samples were previously published [43, 44]. Experimental protocols were approved by the Erasmus MC Medical Ethics Committee following the Medical Research Involving Human Subjects Act.

For usage on Exon Arrays, 12 NAP and 8 PCa samples were obtained via radical prostatectomies (RP) and histologically evaluated by an uropathologist after haematoxylin/eosin staining of tissue sections. 10 cancer samples obtained by TURP and 12 LNPCa samples obtained via lymphadenectomy were also added to the cohort.

For quantitative real-time RT-PCR, an additional 40 PCa, 43 TURP, 1 LNPCa and 5 NAP samples were chosen along with 3 PCa-negative TURP and 2 lymph node samples that served as controls (see Supplementary Table 2).

### Hybridization of exon arrays for clinical samples from normal adjacent prostate

RNA isolation from snap-frozen PCa and NAP samples was performed using RNAbee (Campro Scientific, Berlin, Germany). GeneChip Human Exon 1.0 ST arrays (Affymetrix, Santa Clara, CA, USA) were used to determine expression profiles of each sample. Experiments were performed at the Center for Biomics, Erasmus MC, Rotterdam, the Netherlands and at ServiceXS, Leiden, the Netherlands, according to the manufacturer's instructions [27].

### Discovery of novel prostate cancer-associated transcripts

All datasets were normalized via RMA as implemented in the aroma.affymetrix Bioconductor R-package ([45]; CDF used: HuEx-1_0-st-v2,fullR3,A20071112,EP.CDF, see http://www.aroma-project.org/) and summarized transcript cluster (TC) expression values were obtained for the “full” evidence level. An adapted COPA [22] was performed on log2 expression values and a threshold of $\frac{2\cdot \;MAD\;(t\,r\,a\,n\,s\,c\,r\,i\,p\,t\;c\,l\,u\,s\,t\,e\,r\;z-s\,c\,o\,r\,e\,s)}{0.6745}$ was used to detect outlier samples (as suggested by [46, 47]). TCs with known gene assignment based on Affymetrix NetAffx annotation (NA32, based on hg19), outliers in normal tissue samples or less than 5% outliers in cancer samples were removed. All remaining TCs were grouped based on proximity (less than 250 kb apart), same strand and similarity in outlier profile (Spearman's *p* ≥ 0.5), after which the combined TCs are referred to as EPCATs (see Figure 1). EPCATs that were detected in only one dataset or that comprised less than 12 physical probes on the array were removed. In case EPCATs differed between datasets, all involved TCs were merged into a single EPCAT in order to maximize size and complete the transcript.

### Independent validation via RNA-seq data

Independent validation was performed using RNA-seq data of 27 organ-confined PCa samples from 18 patients obtained via laser capture micro dissection and 5 LNPCa samples. RNA-sequencing was performed on a Genome Analyzer II platform using TruSeq adapters (Illumina, San Diego, CA, USA) at Aros Applied Biosciences (Aarhus, Denmark). Sequencing reads were aligned to a pre-indexed hg19 human reference genome using TopHat 2.0.4 [48]. Resulting BAM files were pooled based on tissue type (PCa and LNPCa) to increase resolution for less abundant transcripts and genomic regions covered by EPCATs including 10 kb flanks were extracted. Cufflinks 2.0.2 was executed in reference guided fashion [26, 49] and results were curated manually using IGV [50], linking single exons into transcripts and further adding candidates that were missed by Cufflinks. Curated exon-intron boundaries were used to design junction spanning PCR primers.

### cDNA synthesis and RT-PCR analysis

RNA-Bee reagent (Campro Scientific, Veenendaal, The Netherlands) was used for total RNA isolation according to manufacturer's protocol. RNA quality was checked on 1% agarose gel and cDNA was synthesized using MMLV-reverse transcriptase kit, according to manufacturer's instructions. EPCAT expression was validated in 6 cell lines (VCaP, 22RV1, LNCaP, PC3, PC346c, DU145 [51–56]) using RT-PCR. Custom PCR primers and TaqMan probes were designed using Primer 3 [57]. Primers were ordered by Sigma Aldrich (St. Louis, MO, USA), probes were ordered at IBA-Lifesciences (Göttingen, Germany, see Supplementary Tables 9–10). ABsolute QPCR ROX Mix from Thermo Scientific (Waltham, MA, USA) was used to perform TaqMan real-time PCR analysis on a 7500 Fast Real-Time PCR System from Applied Biosystems (Foster City, CA, USA). Two housekeeping genes, GAPDH (assay ID Hs99999905_m1, Applied Biosystems Foster City, CA, USA) and HMBS were used as endogenous references and a mixture of cDNAs from prostate carcinoma xenografts as calibrator. Quantification of HMBS was performed using 0.33 μM of primer solution (forward: 5′ CATGTCTGGTAACGGCAATG 3′ and reverse: 5′ GTACGAGGCTTTCAATGTTG 3′) in Power SybrGreen PCR Master Mix (Applied Biosystems), according to thermocycling protocol recommended by the manufacturer. Transcript quantities for each sample were normalized against the average of two endogenous references and relative to a calibrator.

### Determining full length sequences of novel transcripts

RT-PCR validated exons were Sanger sequenced using ABI Prism BigDye Terminator v3.1 Ready Reaction Cycle Sequencing Kit. After PCR processing, samples were analyzed using ABI Prism 3100 Genetic Analyzer (Applied Biosystems, Foster City, California, United States).

To identify the 5′ and 3′ ends of PCR-validated EPCATs, a nested primer approach was used on a λgt11 full length cDNA library of the LNCaP prostate cancer cell line. The λgt11 outer primers were: 5′ TTCAACATCAGCCGCTACA 3′ (forward) and 5′ AAATCCATTGTACTGCCGGA 3′ (reverse). The λgt11 inner primers were: 5′ ACTGATGGAAACCAGCCATC 3′ (forward) and 5′ CCGTATTTCGCTAAGGAAA 3′ (reverse). For amplification of the 5′ end of an EPCAT, 0.15 μl of outer forward λgt11 primer and 0.15 μl outer reverse EPCAT primer were used. For amplification of the 3′ end of an EPCAT, 0.15 μl of the outer reverse λgt11 primer and 0.15 μl outer forward EPCAT primer were used. The first reaction template was a 1:10 diluted λgt11 cDNA library preheated to 95°C for 5 minutes. For the second reaction, all quantities were doubled and inner primers as well as 1 μl of PCR product from first reaction were used. PCR products were loaded on 1% agarose gel in 1x TBE and the specific band was extracted using GeneJETGel extraction kit (Thermo Fisher Scientific Inc, Waltham, Massachusetts) following manufacturer's instructions. Specific products were directly used for sequencing and product concentration was determined using a Nanodrop Spectrophotometer ND-1000 (Thermo Fisher Scientific Inc, Waltham, Massachusetts). Sequencing reaction was the same as for RT-PCR products.

### Investigation of transcriptional regulation of EPCATs

Androgen regulation of EPCATs was investigated via a public dataset comprising LNCaP cells grown in androgen depleted medium (DCC) or in 10 nM R1881 supplemented medium (GSE32875, [36]). Averaged log2 transformed expression values of all TCs for each EPCAT were used for all analyses. Welch's t-test was used for comparison of both conditions and *p*-values were corrected using Benjamini & Hochberg [58]. ERG and ETV1 regulation was evaluated using Spearman's correlation coefficient. AR and ERG binding in EPCAT regions was further investigated using public ChIP-seq data [25]. Peaks called by Yu *et al*. were converted to hg19 using liftOver (https://genome.ucsc.edu/cgi-bin/hgLiftOver) and overlapped with previously identified candidate EPCATs via bedtools [59] including 50 kb flanks. Potential regulation was assumed if at least one peak was falling into the candidate region. For coexpression analysis of genes overlapping EPCATs on the same strand, genes from the UCSC known genes table were intersected with EPCAT regions using bedtools. HGNC symbols for overlapping genes were obtained via biomaRt [60] and median expression values of associated TCs were correlated with EPCAT expression (Spearman's correlation coefficient).

### Computational evaluation of coding potential

Evaluation of coding potential was performed for hg19 build sequences using iSeeRNA (1.2.1) [28] and PhyloCSF (downloaded 22.11.2013) [29]. For iSeeRNA, all RT-PCR validated exon locations were supplied in BED12 format and known coding genes retrieved from the UCSC RefSeq table served as positive controls. For PhyloCSF, a FASTA file containing multiple species alignments for each EPCAT was obtained via the Galaxy ‘Stitch Gene blocks’ tool (http://usegalaxy.org/). Alignments were based on a 46 way Multiz alignment of hg19. All genome builds were converted to common names and intersected with a panel of 29 mammals offered by PhyloCSF. After splitting the FASTA file by gene, PhyloCSF was run using options –frame*s* = 3 –aa for each gene. Two known coding genes, GAPDH and ERG, served as controls.

### Computational evaluation of conservation

For each EPCAT's exons, we downloaded base-wise conservation scores (PhyloP) based on Multiz alignments of 100 vertebrates from the UCSC Genome Browser (http://genome.ucsc.edu). Per EPCAT, PhyloP basewise scores were averaged in 50 bp windows and the highest of these averages was used as overall representative score of the gene locus. 1000 randomly selected coding RefSeq genes as well as 1000 randomly selected Repetitive elements (RepeatMasker, UCSC Genome Browser) served as controls.

### Tissue microarray construction

A total of four tissue microarrays (TMAs) was used to evaluate expression of two EPCATs (EPCAT4R966 and EPCAT2F176) in patient tissues, xenografts and cell lines (see Supplementary Tables 5a–5b).

The first TMA consisted of 481 patient samples from radical prostatectomies for PCa and several control specimens as described previously [61]. Controls comprised normal prostate tissues from radical cystoprostatectomies (RCP, *n* = 7), urothelial cell carcinomas (*n* = 5), invasive ductal mammary adenocarcinomas (*n* = 5), palliative transurethral resection of the prostate (TURP, *n* = 10), prostate cancer lymph node metastasis (LNPCa, *n* = 10) and placenta (*n* = 1). Additionally, PCa cell lines (*n* = 7) and prostate cancer xenografts models (*n* = 22) were included.

The second TMA, comprised 127 triplicate patient samples of nonneoplastic prostate tissue. We performed a search in PALGA (Pathologisch anatomisch landelijk geautomatiseerd archief, Houten, the Netherlands) and selected 53 patients who had undergone RCP or pelvic exenteration (PE), due to bladder cancer. TURP samples from 74 patients with clinical BPH were included in the TMA as well. All operations had taken place between 2003 and 2013. In RCP and PE specimen, we selected prostate glands from the peripheral zone, whereas transition zone was selected in TURP samples. All slides were histopathologically reviewed to exclude presence of prostate adenocarcinoma. Several tissues were added to the TMA as landmarks: placenta (*n* = 1), kidney (*n* = 1), ovary (*n* = 1) and spleen (*n* = 1).

The third TMA contained 119 LNPCa samples from patients who underwent RP combined with a lymph node exploration, obtained between 1989 and 2006 at the Erasmus MC.

The fourth TMA comprised a total of 120 PCa samples, operated between 1982 and 2009 in the Erasmus MC. 35 samples were obtained after RP and 85 samples contained TURP material. 65 of 120 patients were hormone refractory prostate cancers (CRPC), 55 patients were hormone sensitive. After patient selection, all TMAs were constructed using an automated TMA constructor (ATA-27 Beecher Instruments, Sun Prairie, WI, USA) available at the Department of Pathology, Erasmus MC.

### *In situ* hybridisation and quantification - RNAscope

RNA *in situ* hybridisation on FFPE tissue was performed with RNAscope (Advanced Cell Diagnostics, Inc, Hayward, California). One week old 5 μm sections were dewaxed and treated with heat and protease antigen retrieval according to manufacturer's protocol. Specific target probes for EPCAT2F176 (targeting 466 nt) and EPCAT4R966 (targeting 1152 nt) provided by Advanced Cell Diagnostics were hybridized on the tissue (see Supplementary Table 8 for EPCAT sequences). Signal amplification on the probe was followed by visualisation with fast-red and counterstaining with haematoxylin. Probes for housekeeping gene ubiquitin C and bacterial gene dapB served as positive and negative controls. Scoring of TMAs was performed in-house by a trained uropathologist. Only counts above 0 were considered as positive.

### Assessment of diagnostic potential

Diagnostic potential was assessed by creating a receiver operator characteristic for 11 EPCATs for which working TaqMan probes were available. Samples that were present in the EMC Exon Array dataset were used as discovery cohort, while the remaining 47 samples (40 PCa, 5 NAP) were used for validation. The R package ‘optAUC’ was used for AUC maximization in the test cohort and ROC-curves were created using the ‘ROC’-package.

### Kaplan-Meier survival analysis and evaluation of prognostic potential

Samples of localized PCa from the ‘EMC’ dataset were used to determine prognostic potential of the 15 validated EPCATs. For each EPCAT, TC intensity values were averaged and used as representative measures of gene expression. Partition Around Medoids (PAM, R-package ‘cluster’) was used to define two groups of samples with high and low expression of an EPCAT. Overrepresentation of three clinical endpoints was evaluated for 54 patients with available clinical information using a bootstrapping approach. The clinical endpoints were: (i) biochemical recurrence, defined as a rise in serum PSA level from undetectable to ≥ 0.2 ng/ml in at least two consecutive measurements (at least three months apart) after RP; (ii) clinical progression, defined by occurrence of metastasis in lymph nodes or other organs (iii) prostate cancer related death. For bootstrapping, class labels (clinical endpoints of patients) were permuted, sampled and assigned to two groups with PAM defined sizes. Sampling was repeated 10,000 times for each EPCAT to create a sample distribution and *p*-values were calculated as the number of samplings having more positive associations with a clinical endpoint than the original EPCAT entry, divided by the number of iterations. In addition, Kaplan-Meier curves (R package ‘survival’) were created for each EPCAT and clinical endpoint.

### siRNA knockdown and cell viability

Silencer Select siRNA probes were designed by and purchased from Ambion (Life Technologies, Carlsbad, CA, USA). SiRNA probes consisted of a sense and an antisense siRNA for each target transcript with the following sequences:

EPCAT1F273: GGGAAGCAUUGAAAUAGUAtt (sense siRNA), UACUAUUUCAAUGCUUCCCag (antisense siRNA); EPCAT3R522: CAGCUAAGCUGAAAAAGCAtt (sense siRNA), UGCUUUUUCAGCUUAGCUGtc (antisense siRNA); EPCAT4R966: GGCUUGUCGUGUGAUCUAAtt (sense siRNA), UUAGAUCACACGACAAGCCta (antisense siRNA); EPCAT8R190: CCAUGUCCUUGAGAUAAAAtt (sense siRNA), UUUUAUCUCAAGGACAUGGga (antisense siRNA); EPCAT15F850: GAAUGAGAGUCAUCA UGUAtt (sense siRNA), UACAUGAUGACUCU CAUUCag (antisense siRNA); EPCATXR234: CCUUAACAAUGGAUCUGCAtt (sense siRNA), UGCAGAUCCAUUGUUAAGGtt (antisense). PCa cells LNCaP (12*10^3^ cells) and 22RV1 (8*10^3^ cells) were transferred to 96 wells plates and kept in RPMI 1640 and 5% FCS. After one day, cells were transfected in triplicate with 500 nM siRNA using DharmaFECT 3 Transfection Reagent (GE Healthcare, Little Chalfont, UK) according to the manufacturers' instructions (20 μl siRNA mix and 80 μl 5% DCC medium per well). 100 μl 5% FCS medium was added to all wells not measured at day 0. Proliferation was subsequently measured using 3-(4,5-dimethylthiazol-2-yl)-2,5-diphenyl tetrazolium bromide (MTT) at indicated time points (LNCaP: 0, 5, 8 days; 22RV1: 0, 4, 7 days). All experiments were performed twice.

## SUPPLEMENTARY FIGURES AND TABLES






