# Supplementary material for: Novel long non-coding RNAs are specific diagnostic and prognostic markers for prostate cancer
Source: Oncotarget. 2015 Feb 17;6(6):4036–50. doi: 10.18632/oncotarget.2879 (PMC4414171; doi:10.18632/oncotarget.2879)
Supplement: Supplementary file 1 [file oncotarget-06-4036-s001.pdf]

SUPPLEMENTARY FIGURES AND TABLES

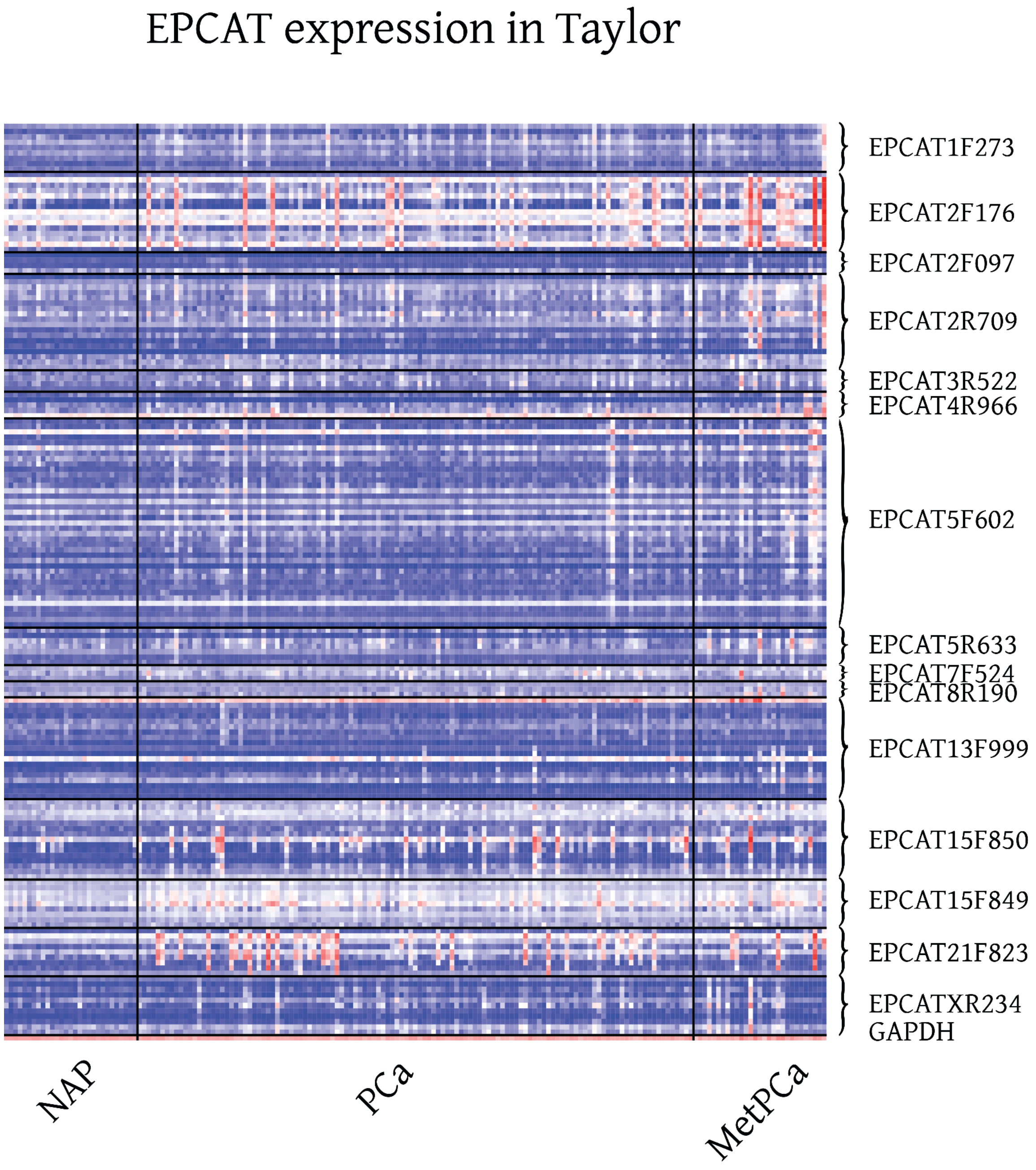

Supplementary Figure 1: Expression of RT-PCR validated EPCATs in samples of 'Taylor' (GSE21034), comprising localized prostate cancer (PCa), metastasis (MetPCa) and normal adjacent prostate (NAP) tissue.

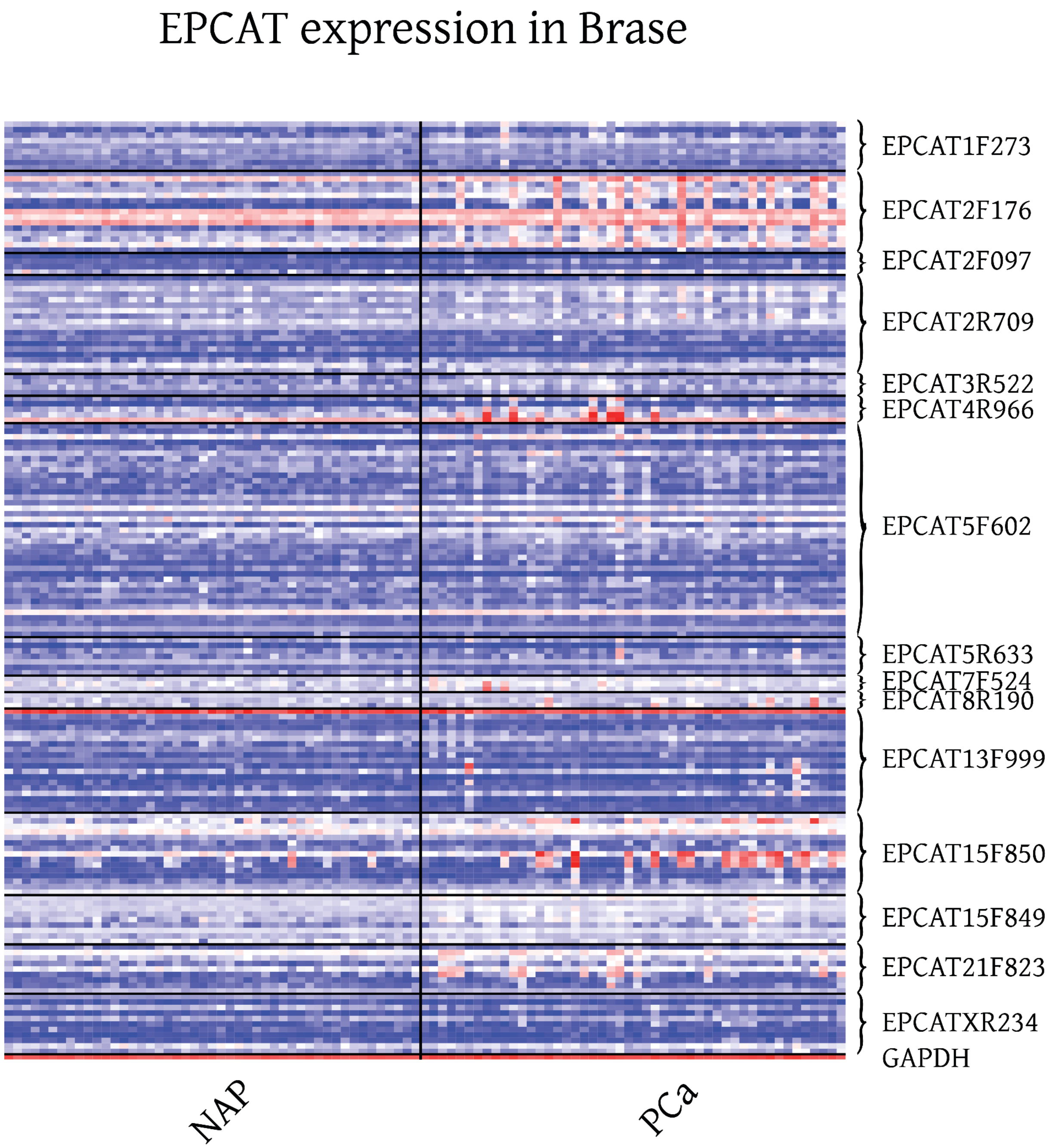

Supplementary Figure 2: Expression of RT-PCR validated EPCATs in 'Brase' (GSE29079), comprising localized prostate cancer (PCa) and normal adjacent prostate (NAP) tissue.

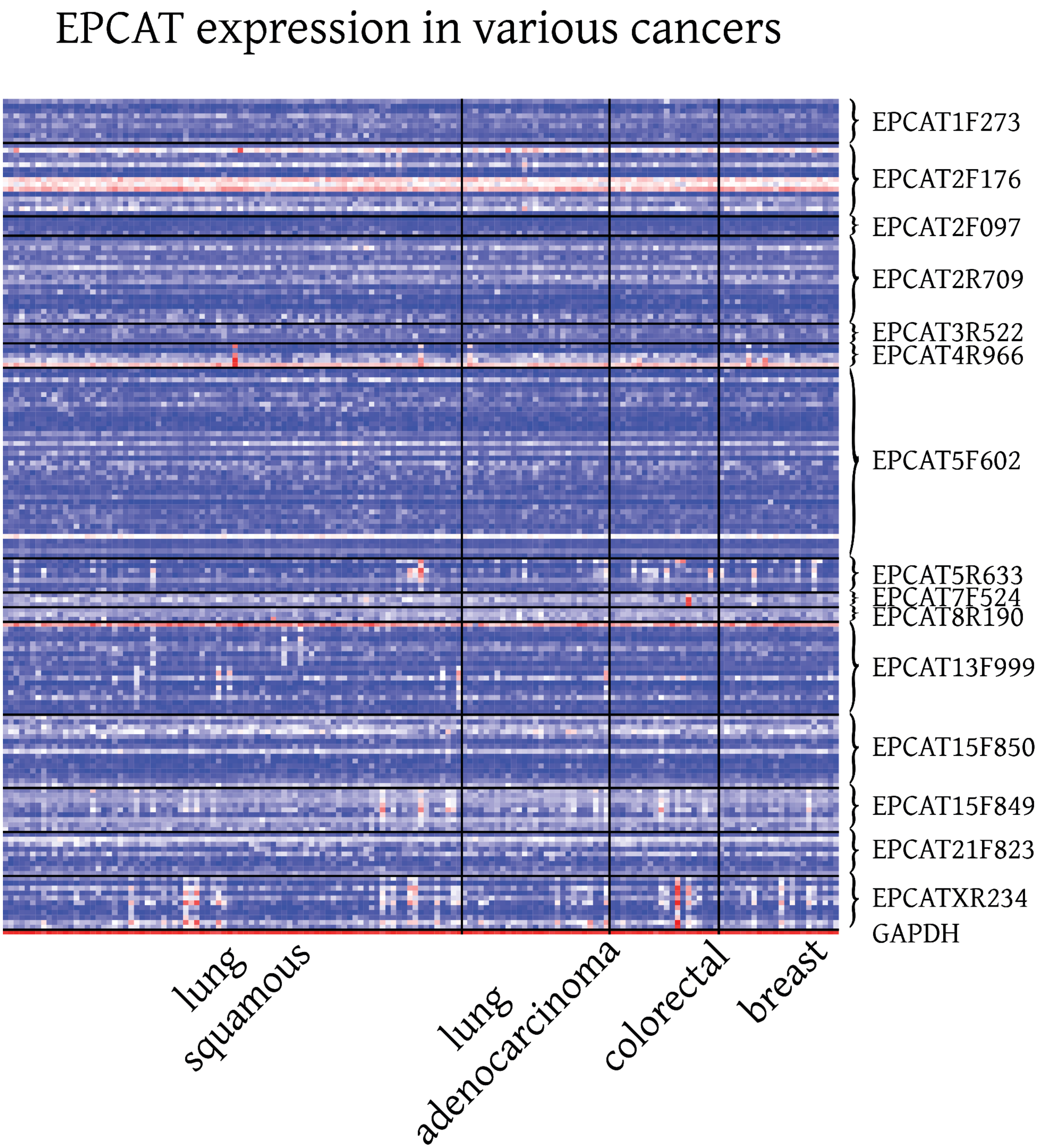

Supplementary Figure 3: Expression of RT-PCR validated EPCATs in breast, colorectal and lung cancer tissue tissue (GSE21034).

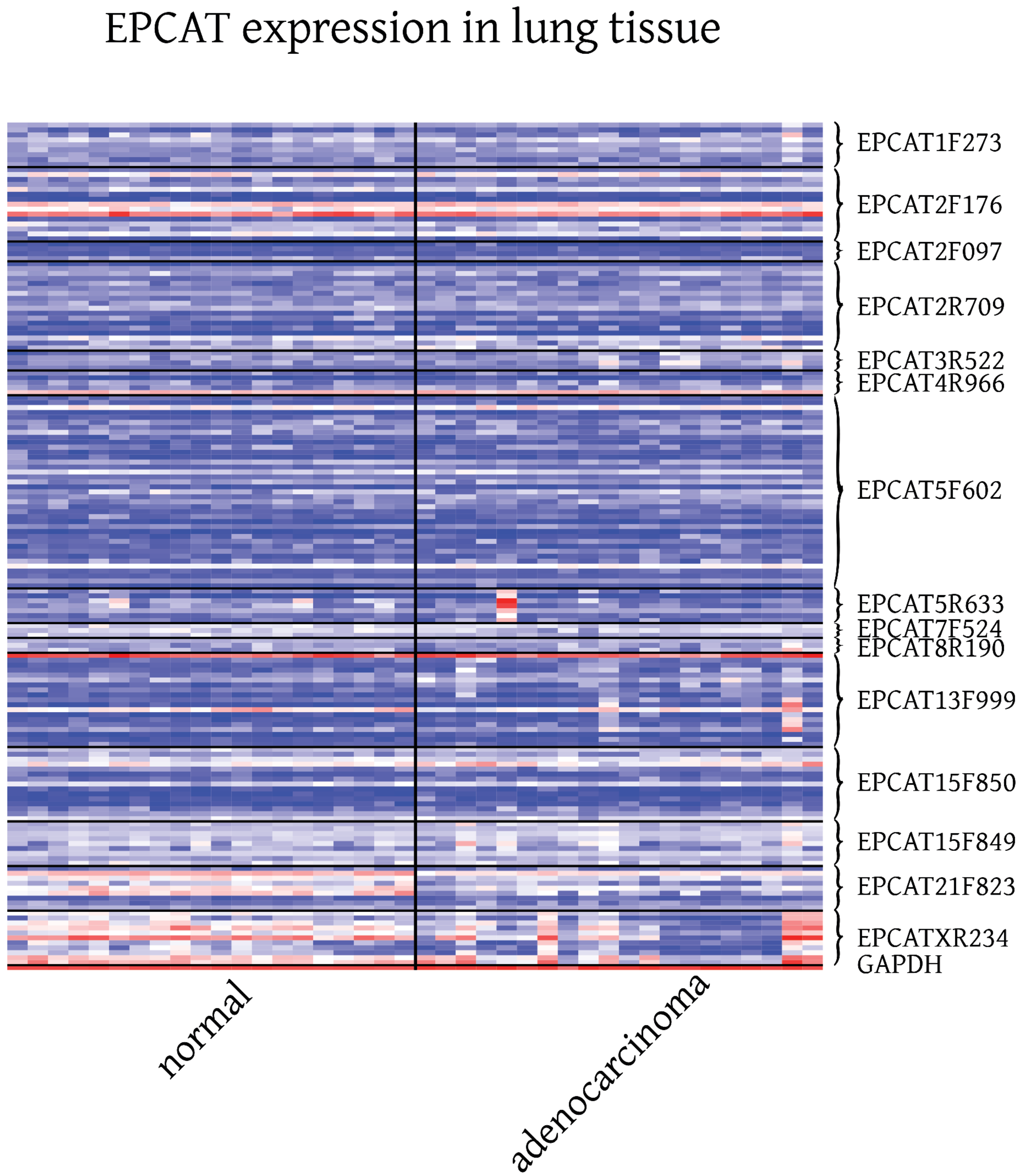

Supplementary Figure 4: Expression of RT-PCR validated EPCATs in lung cancer (GSE12236).

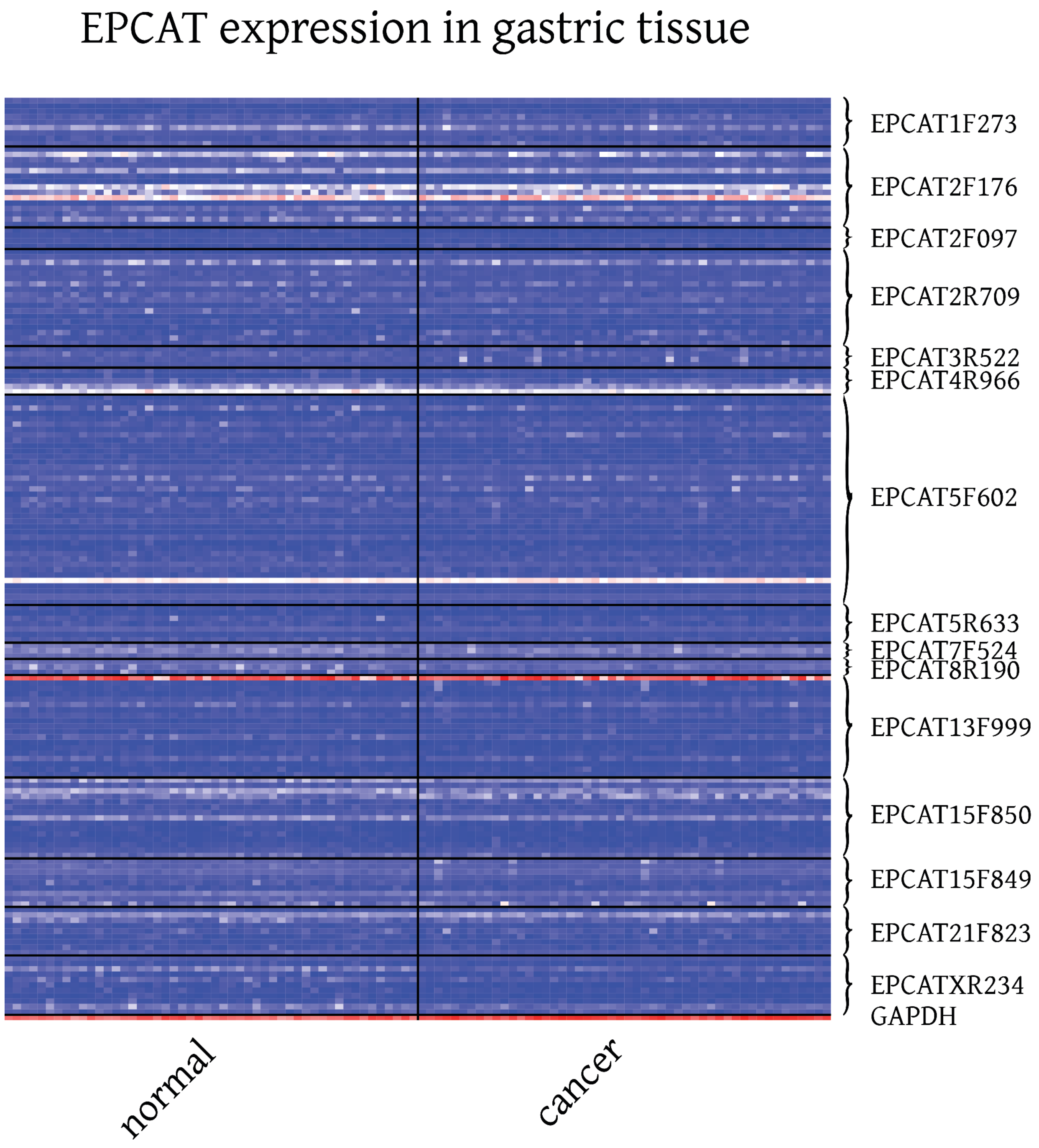

Supplementary Figure 5: Expression of RT-PCR validated EPCATs in gastric cancer (GSE13195).

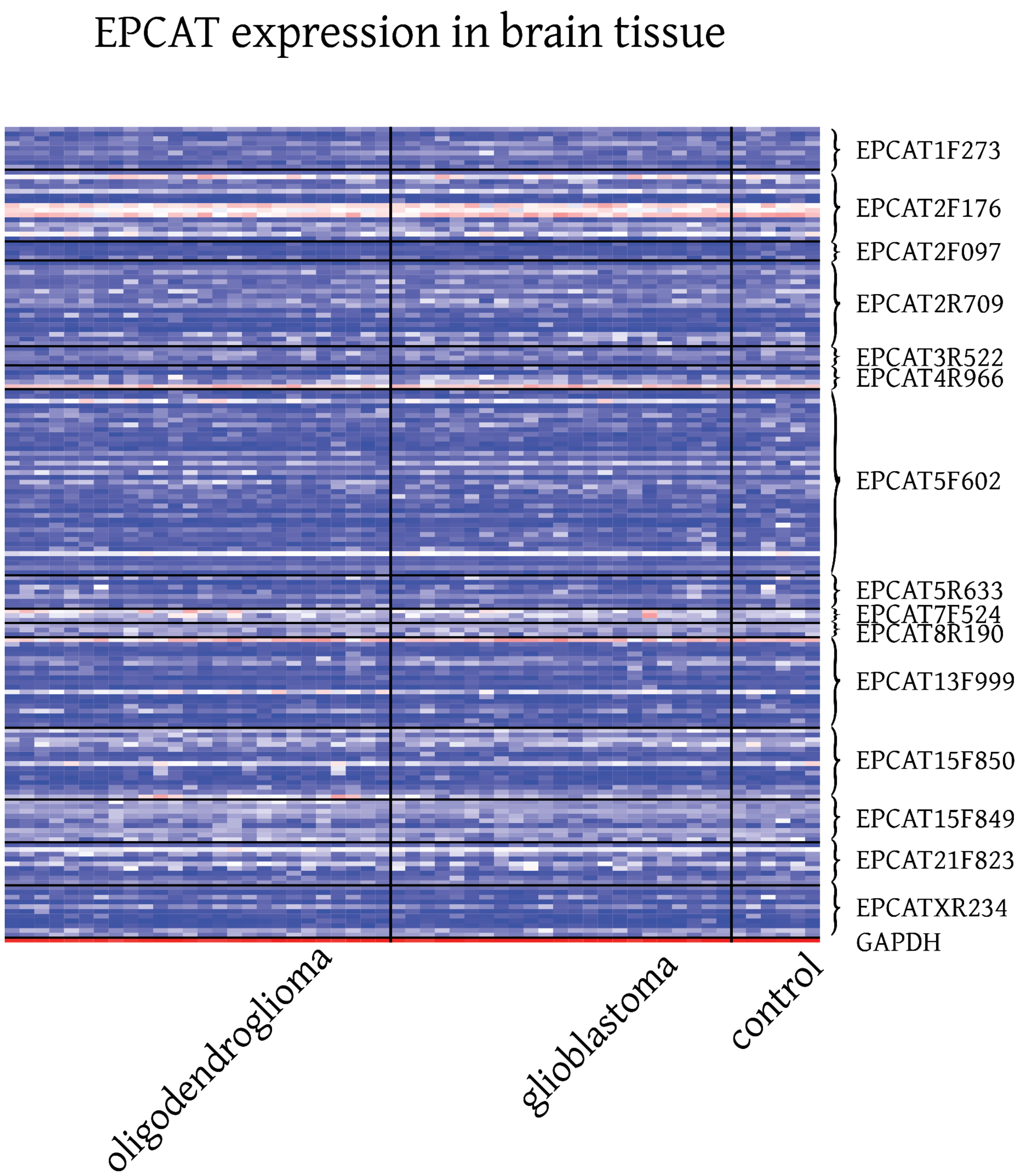

Supplementary Figure 6: Expression of RT-PCR validated EPCATs in brain cancer (GSE9385).

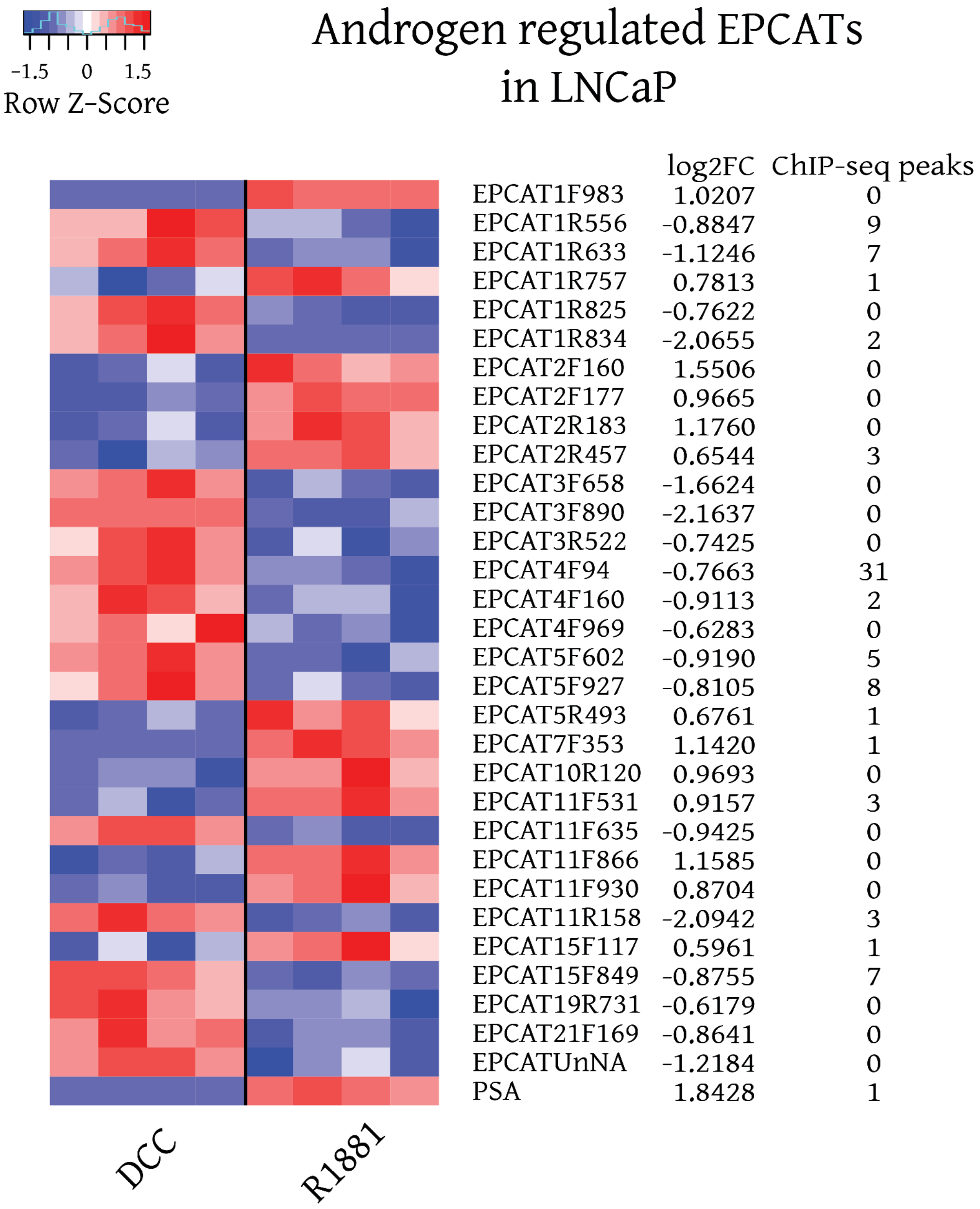

Supplementary Figure 7: Expression of potentially AR regulated EPCATs in prostate cancer cell line LNCaP, treated with a synthetic androgen (R1881) or grown in androgen free medium (DCC) (GSE32875). AR association was evaluated using Welch's t-test, only EPCATs showing significant differences ($p < 0.05$) and with an expression in- or decrease of at least 50% were included.

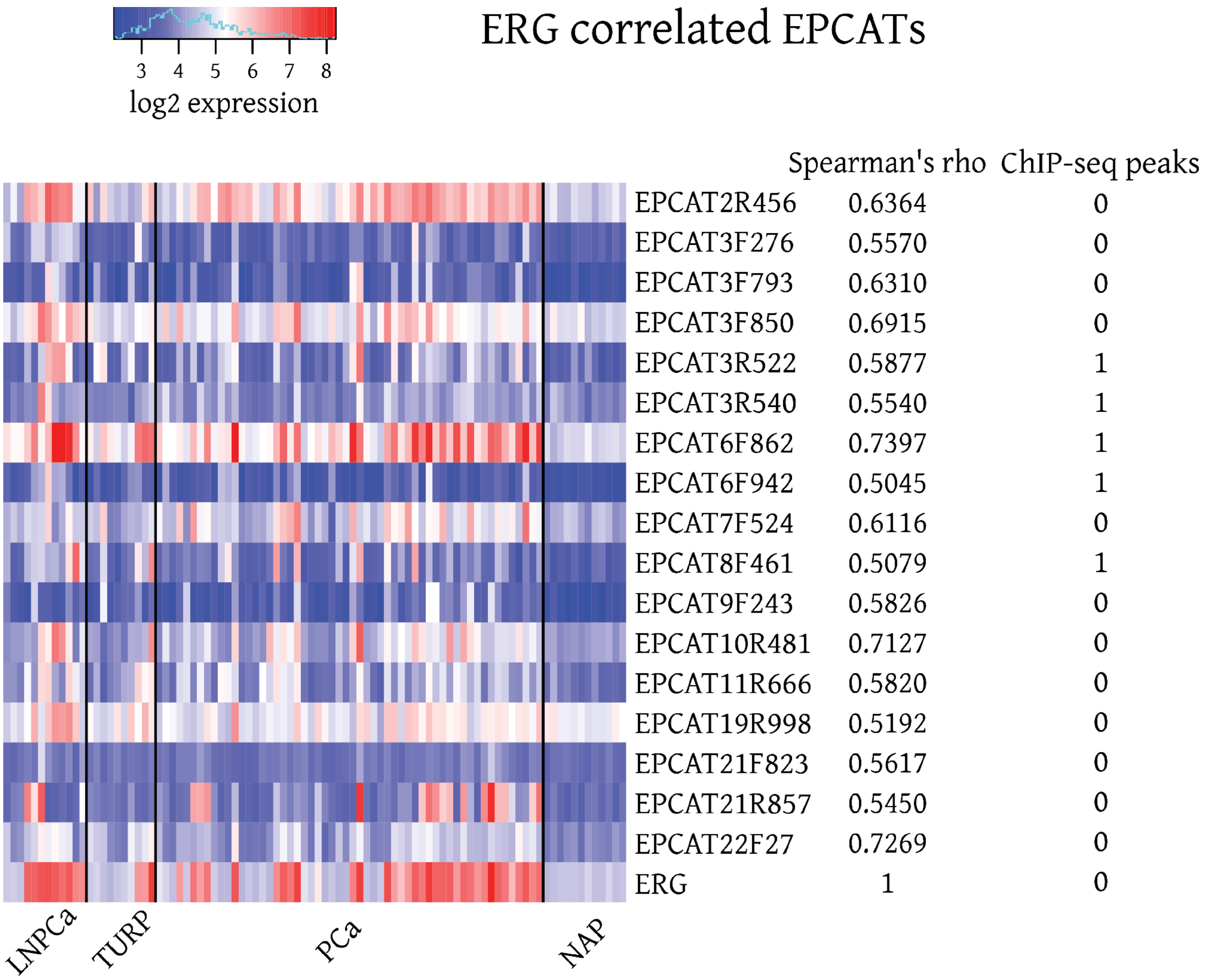

Supplementary Figure 8: Expression of potentially ERG regulated EPCATs in the EMC dataset. ERG association was evaluated using Spearman’s correlation coefficient, only EPCATs showing significant correlation (rho > 0.5) were included.

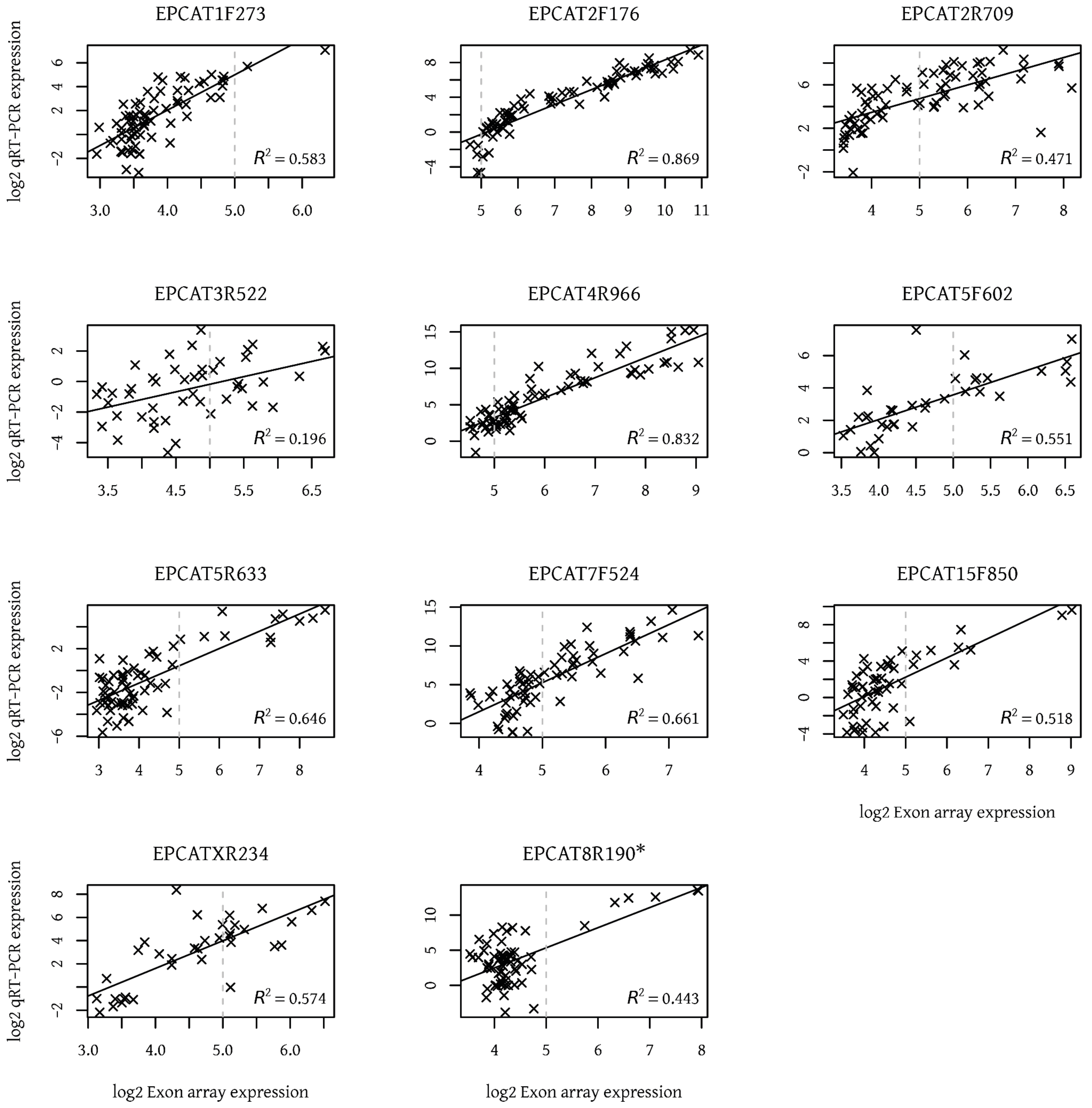

Supplementary Figure 9: Comparison of EPCAT expression measurements between Affymetrix Exon Arrays and qRT-PCR normalized against GAPDH and HMBS for 78 samples of the 'EMC' dataset. Samples that showed low expression in EMC but no expression for qRT-PCR were excluded (varying for each EPCAT).

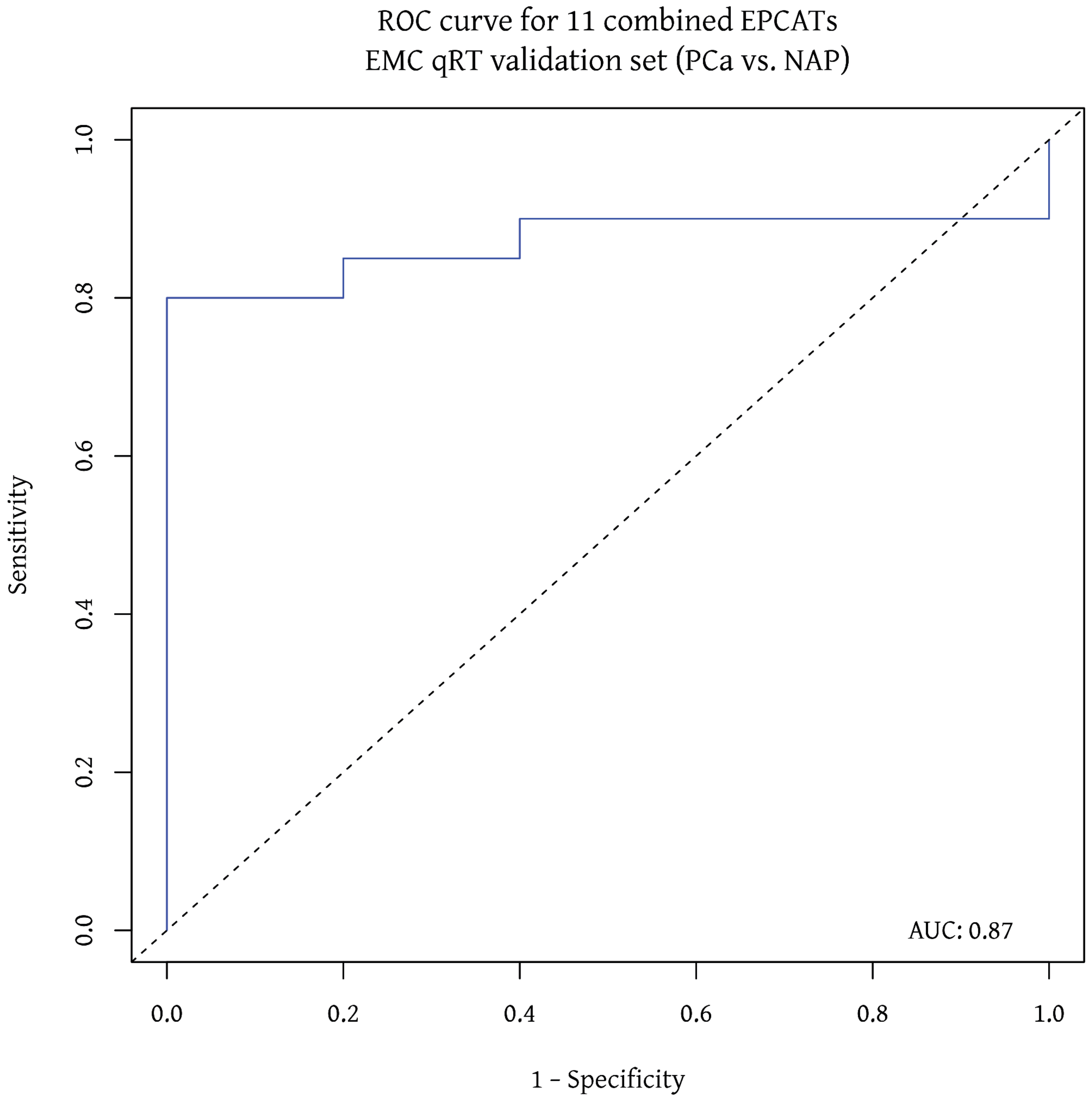

Supplementary Figure 10: ROC curve for distinguishing PCa and NAP samples by qRT-PCR. A diagnostic panel was created by combination of the 11 validated EPCATs on an independent patient cohort tested by qRT-PCR.

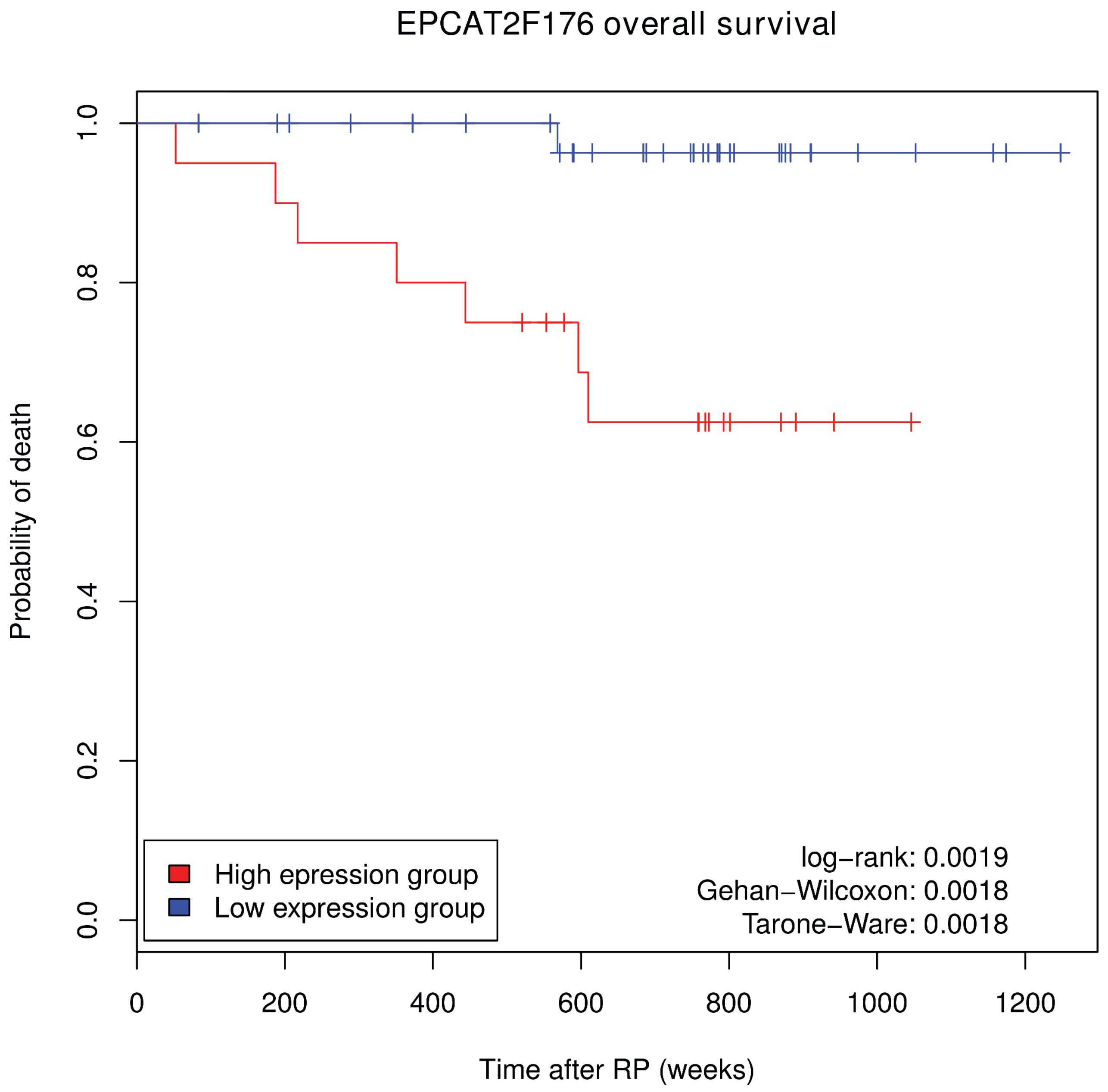

Supplementary Figure 11: Kaplan-Meier curve of overall survival for EPCAT2F176. Groups of high and low expression were defined by unsupervised clustering of Exon Array expression values using PAM.

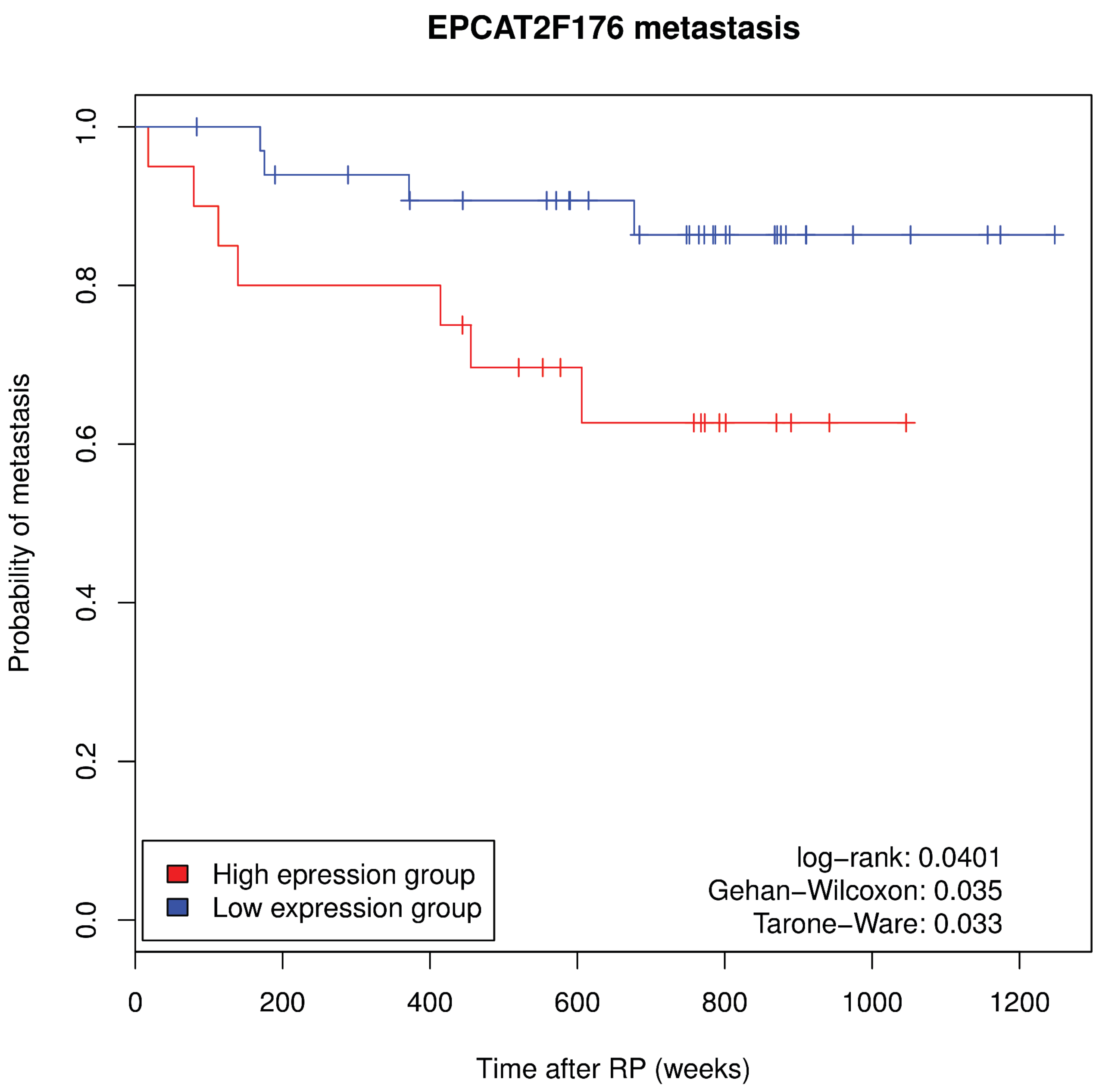

Supplementary Figure 12: Kaplan-Meier curve of metastasis free survival for EPCAT2F176. Groups of high and low expression were defined by unsupervised clustering of Exon Array expression values using PAM.

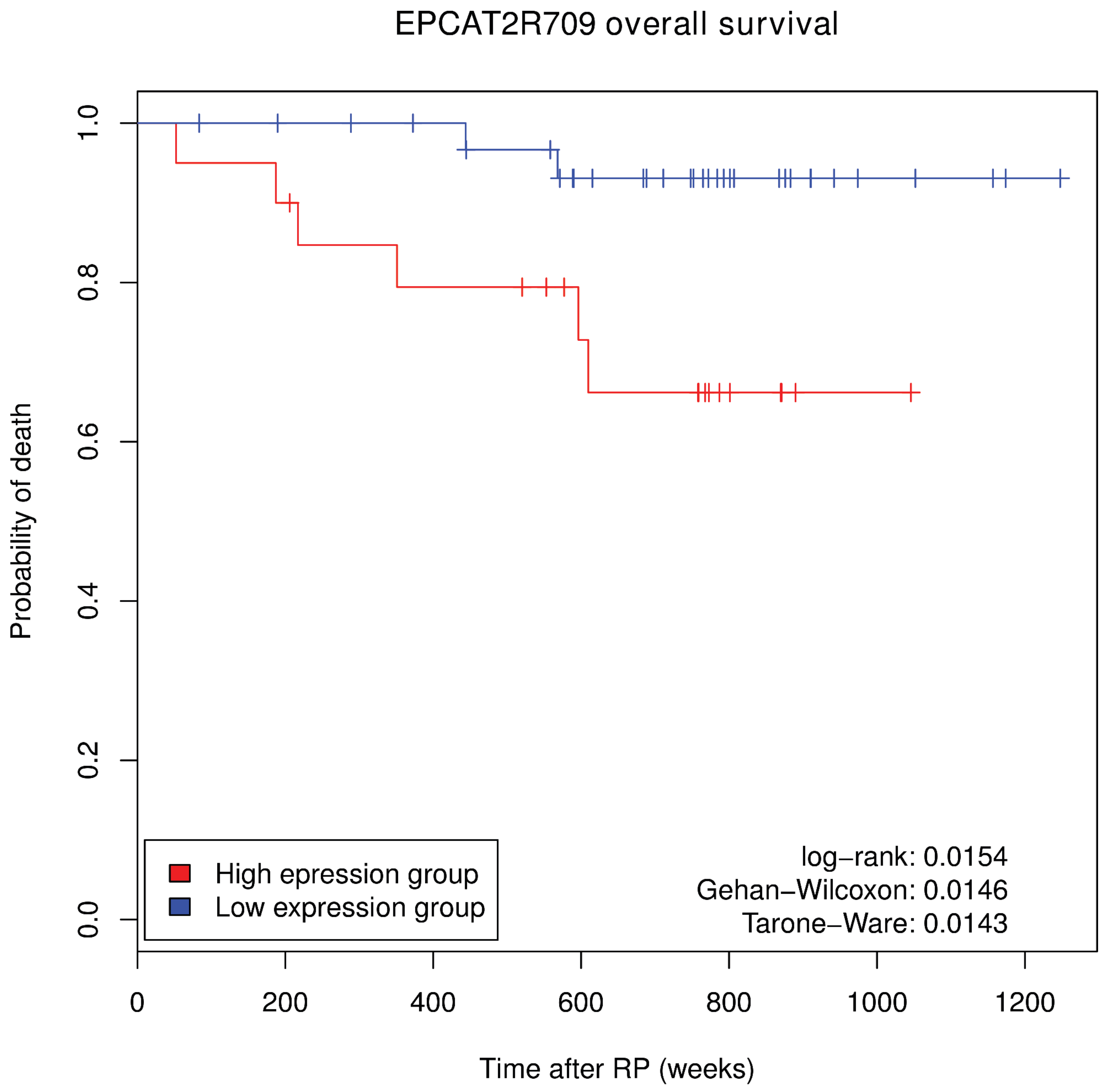

Supplementary Figure 13: Kaplan-Meier curve of overall survival for EPCAT2R709. Groups of high and low expression were defined by unsupervised clustering of Exon Array expression values using PAM.

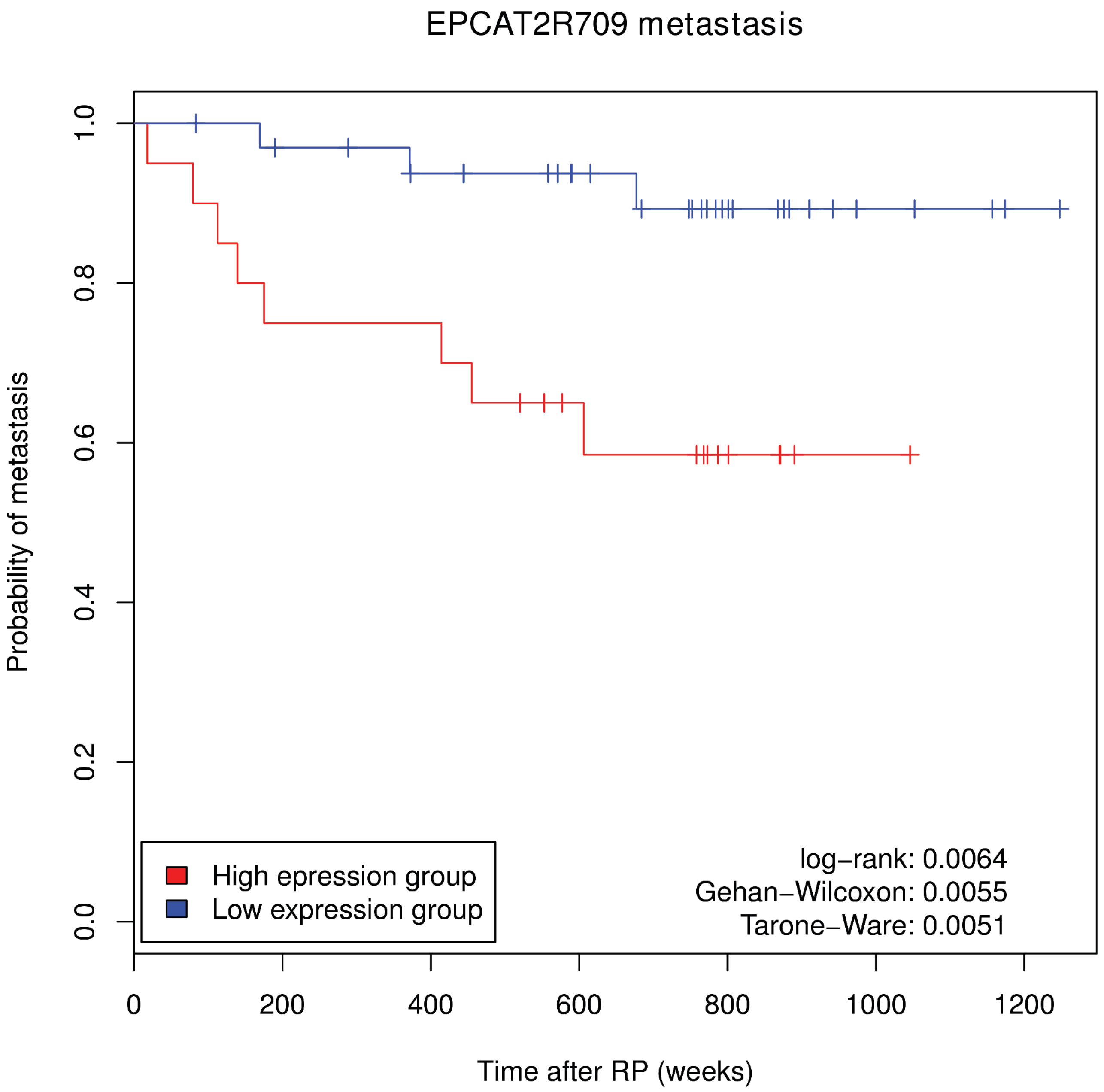

Supplementary Figure 14: Kaplan-Meier curve of metastasis survival for EPCAT2R709. Groups of high and low expression were defined by unsupervised clustering of Exon Array expression values using PAM.

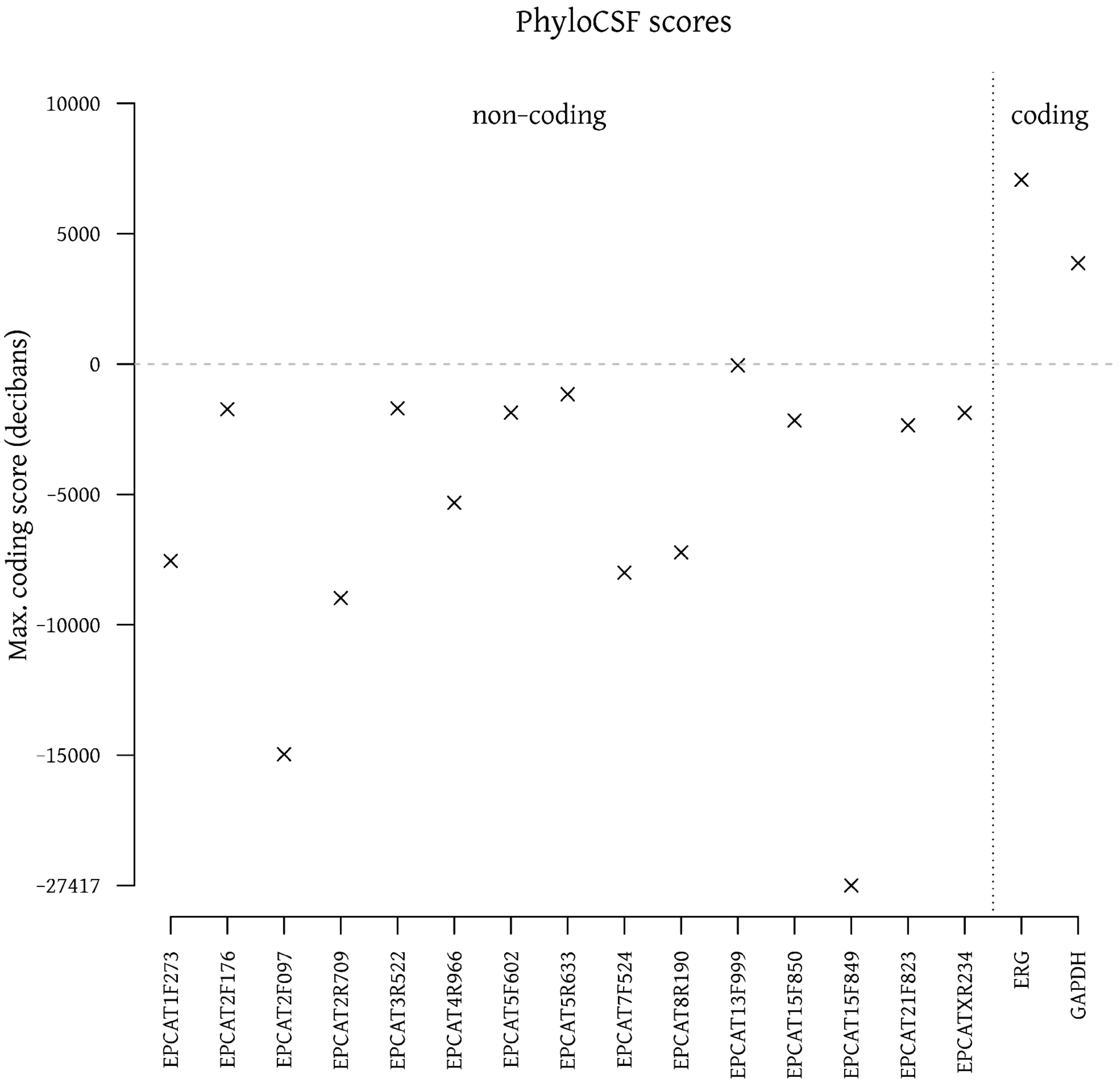

Supplementary Figure 15: Evaluation of coding potential. Sequences of RT-PCR validated EPCATs as well as two control genes (ERG and GAPDH) were processed with PhyloCSF after obtaining 46 way Multiz alignments to hg19 from Galaxy's 'Stitch Gene blocks' tool (http://usegalaxy.org/). All EPCATs scored below zero, indicating no protein coding potential.

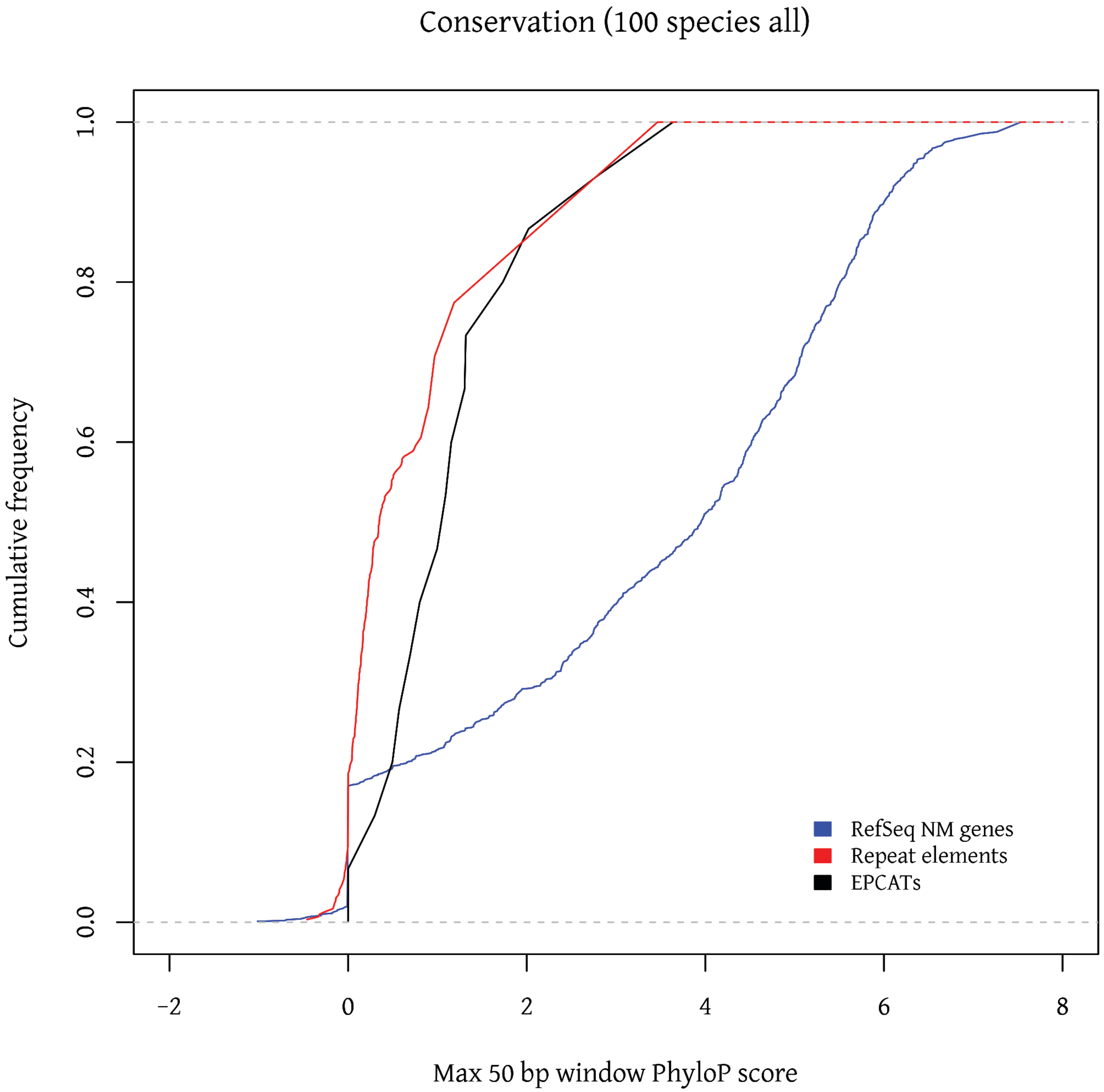

Supplementary Figure 16: Comparison of sequence conservation of exons. 15 RT-PCR validated EPCATs (black), 1000 protein coding (NM) RefSeq genes (blue) and 1000 randomly selected repeat elements (red) were selected. For each gene, PhyloP scores obtained from UCSC were averaged for each 50 bp window and the maximum average was used as total score.

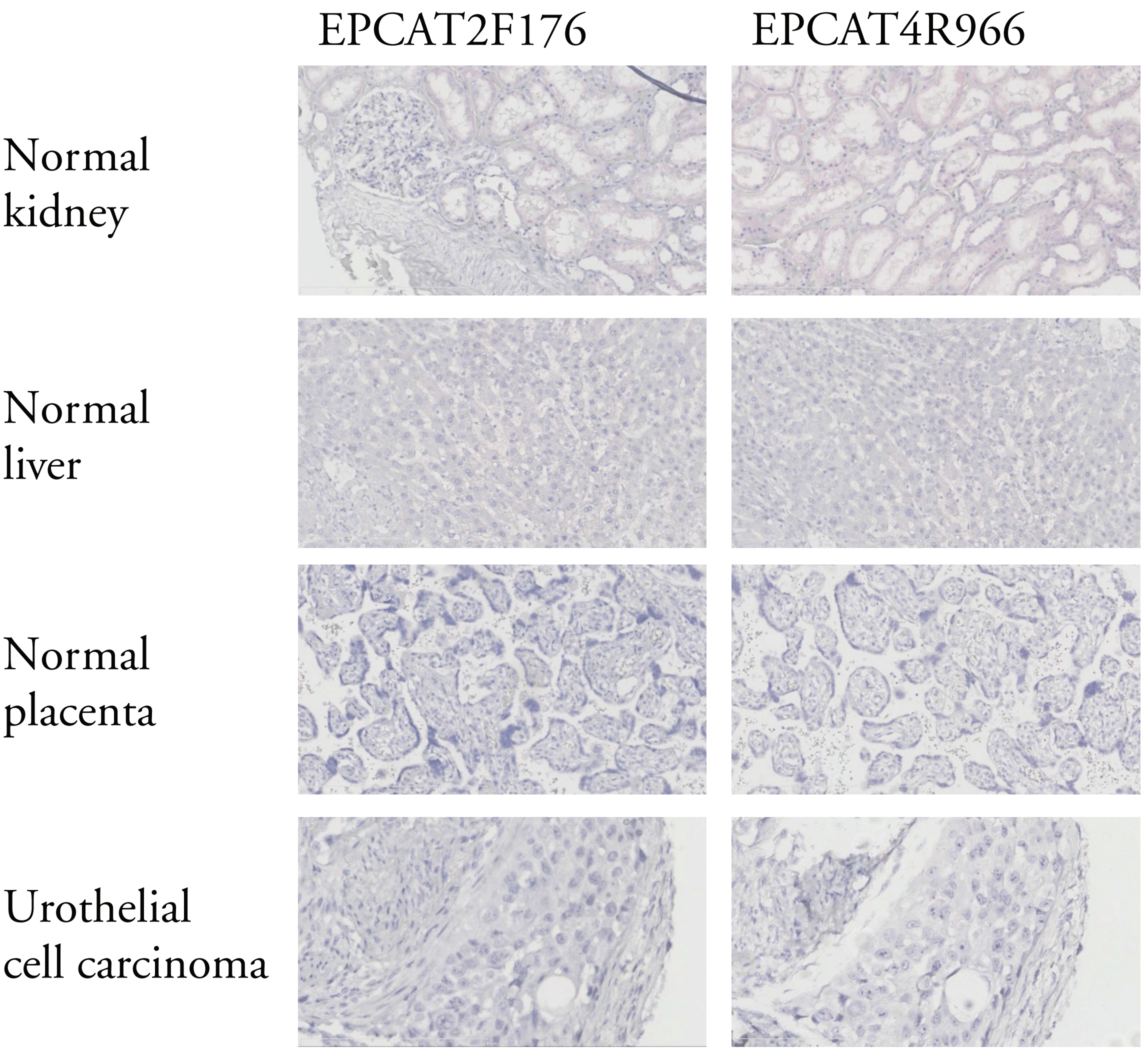

Supplementary Figure 17: *In situ* hybridization of EPCAT2F176 and EPCAT4R966 in various control tissues (200× magnification). All control samples were scored negative, highlighting the PCa-specific expression of both transcripts.

Supplementary Table 1: List of genomic locations of all EPCATs, as well as involved Affymetrix Human Exon Array transcript cluster IDs

Supplementary Table 2: Number of samples per clinical group for each Exon Array dataset (EMC, Taylor, Brase) and both qRT-PCR cohorts

	EMC	Taylor	Brase	qRT discovery	qRT validation
**NAP**	12 (13%)	29 (16%)	48 (51%)	12 (16%)	5 (5%)
**PCa**	56 (63%)	131 (73%)	47 (49%)	43 (57%)	40 (43%)
**TURP**	10 (11%)	-	-	9 (12%)	43 (46%)
**MetPCa**	-	12 (7%)	-	-	-
**LNPCa**	12 (13%)	7 (4%)	-	11 (15%)	1 (1%)
**TURP control**	-	-	-	-	3 (3%)
**LN control**	-	-	-	-	2 (2%)

Supplementary Table 3: List of RT-PCR validated EPCATs in BED12 format, including structural information of exon position and length

Chr	Start	Stop	Name							Exon length	Exon start
chr1	193281156	193868497	EPCAT1F273	0	+	193281156	193868497	0	10	77,138,142,135,552, 109,93,466,96,80	0,3226,37107,39078,43736,145615, 148092,149546,574295,587261
chr2	193261317	193410857	EPCAT2F097	0	+	193261317	193410857	0	12	95,87,99,126,181, 208,130,55,1497, 181,69,165	0,3572,17066,34776,60829,61379 ,61671,69088,70281,73742,7436 5,149375
chr2	181556870	181692235	EPCAT2F176	0	+	181556870	181692235	0	4	299,95,101,267	0,32453,33048,135098
chr2	180981253	181436558	EPCAT2R709	0	-	180981253	181436558	0	8	1050,107,95,273, 118,68,31,119	0,2641,6807,24779,450504,451551, 452604,455186
chr3	80679057	80819475	EPCAT3R522	0	-	80679057	80819475	0	6	349,56,144,156,115,565	0,1325,3909,3909,131552,139853
chr4	169980608	169993351	EPCAT4R966	0	-	169980608	169993351	0	4	714,158,104,172	0,1964,11188,12571
chr5	164775904	165205079	EPCAT5F602	0	+	164775904	165205079	0	3	152,132,97	0,113472,429078
chr5	70642416	70742616	EPCAT5R633	0	-	70642416	70742616	0	4	326,227,144,87	0,7107,99056,100113
chr7	26590351	26594538	EPCAT7F524	0	+	26590351	26594538	0	2	1478,11	0,4077
chr8	54307025	54321348	EPCAT8R190	0	-	54307025	54321348	0	6	1291,82,267,89,97,89	0,1434,5105,7015,11333,14234
chr13	55014817	55015411	EPCAT13F999	0	+	55014817	55015411	0	2	95,1	0,494
chr15	69917267	70112407	EPCAT15F849	0	+	69917267	70112407	0	14	264,197,181,94,145,2863, 72,1230,193,755,138,50,1 095,173	0,3811,46798,52618,59369,70739,7 4885,77981,90658,96666,127450,1 31228,161320,194967
chr15	21082731	21101386	EPCAT15F850	0	+	21082731	21101386	0	3	717,143,3326	0,9611,15329
chr21	39875488	39877234	EPCAT21F823	0	+	39875488	39877234	0	2	425,106	0,164
chrX	93655065	93738198	EPCATXR234	0	-	93655065	93738198	0	4	438,133,1074,3004	0,2022,14508,80129

Supplementary Table 4a: Results of bootstrapping analysis for association of RT-PCR validated EPCAT expression (low vs. high) and clinical outcome based on EMC Exon Array dataset

Bootstrapping term association	EMC *p*-value			Taylor *p*-value		
	PSA-progression	metastasis	PCa-death	PSA-progression	metastasis	PCa-death
EPCAT1F273	0.4570543	0.94020598	0.85891411	0.1266873	1	1
EPCAT2F176	0.5065493	0.04769523	0.00249975	0.7551245	1	1
EPCAT2F097	0.9261074	1	1	0.2769723	0.6457354	1
EPCAT2R709	0.4966503	0.00789921	0.02429757	0.3993601	0.4175582	1
EPCAT3R522	0.6063394	0.59064094	0.85111489	0.9915008	1	1
EPCAT4R966	0.8870113	0.40435956	0.52674733	0.8569143	1	1
EPCAT5F602	0.970303	0.60613939	0.4759524	0.1637836	1	1
EPCAT5R633	0.6109389	0.50454955	0.38276172	0.8578142	0.7067293	1
EPCAT7F524	0.2058794	0.94160584	1	0.2811719	0.490051	1
EPCAT8R190	0.9008099	0.71812819	0.06729327	0.4649535	0.3111689	1
EPCAT13F999	0.3454655	0.19058094	0.57334267	0.7325267	0.7865213	1
EPCAT15F850	0.8153185	1	1	0.4027597	1	1
EPCAT15F849	0.9455054	0.65773423	0.56344366	0.879712	0.5333467	1
EPCAT21F823	0.1814819	0.31556844	0.58714129	0.4242576	0.1250875	1
EPCATXR234	0.5273473	0.92060794	0.99070093	0.7085291	0.590141	1

Supplementary Table 4b: Results of Kaplan-Meier analysis for RT-PCR validated EPCAT expression (low vs. high) and association with biochemical recurrence/PSA-progression based on EMC Exon Array dataset

Kaplan-Meier PSA-progression	Log-rank	Gehan-Wilcoxon	Tarone-Ware
EPCAT1F273	0.822304	0.840066	0.863782
EPCAT2F176	0.55431	0.508751	0.501267
EPCAT2F097	0.355493	0.341533	0.338199
EPCAT2R709	0.65968	0.623281	0.61945
EPCAT3R522	0.906851	0.942022	0.931266
EPCAT4R966	0.479322	0.713114	0.818175
EPCAT5F602	0.229042	0.327943	0.389935
EPCAT5R633	0.492762	0.496482	0.512629
EPCAT7F524	0.45515	0.65948	0.782489
EPCAT8R190	0.509915	0.656146	0.746741
EPCAT13F999	0.220446	0.14333	0.125925
EPCAT15F850	0.747927	0.714793	0.692413
EPCAT15F849	0.207135	0.277104	0.306061
EPCAT21F823	0.31564	0.280606	0.276468
EPCATXR234	0.731864	0.536802	0.446988
pre-operative PSA	0.000019	0.000022	0.000024
pT-stage	0.000446	0.001039	0.001902
Gleason score	0.05253	0.060306	0.065992

Supplementary Table 4c: Results of Kaplan-Meier analysis for RT-PCR validated EPCAT expression (low vs. high) and association with development of clinical metastases based on EMC Exon Array dataset

Kaplan-Meier metastasis	Log-rank	Gehan-Wilcoxon	Tarone-Ware
EPCAT1F273	0.286744	0.283816	0.282656
EPCAT2F176	0.0401	0.035044	0.032952
EPCAT2F097	0.365511	0.367244	0.368998
EPCAT2R709	0.006432	0.005475	0.005127
EPCAT3R522	0.834723	0.76509	0.730381
EPCAT4R966	0.610612	0.606326	0.604812
EPCAT5F602	0.955955	0.995627	0.971211
EPCAT5R633	0.631468	0.695954	0.728961
EPCAT7F524	0.280833	0.28485	0.287111
EPCAT8R190	0.914835	0.987247	0.938661
EPCAT13F999	0.210626	0.228604	0.237991
EPCAT15F850	0.481605	0.482749	0.484056
EPCAT15F849	0.825059	0.82339	0.823735
EPCAT21F823	0.486802	0.477063	0.47403
EPCATXR234	0.302095	0.286277	0.27888
pre-operative PSA	0.000019	0.000022	0.000024
pT-stage	0.09705	0.092336	0.090815
Gleason score	0.005366	0.002211	0.00143

Supplementary Table 4d: Results of Kaplan-Meier analysis for RT-PCR validated EPCAT expression (low vs. high) and association with PCa-related death based on EMC Exon Array dataset

Kaplan-Meier PCa-death	Log-rank	Gehan-Wilcoxon	Tarone-Ware
EPCAT1F273	0.503146	0.488821	0.481851
EPCAT2F176	0.001911	0.001844	0.001826
EPCAT2F097	0.439962	0.440863	0.441762
EPCAT2R709	0.015405	0.014596	0.014256
EPCAT3R522	0.409329	0.369127	0.350838
EPCAT4R966	0.792452	0.787311	0.786213
EPCAT5F602	0.74914	0.819185	0.853669
EPCAT5R633	0.426692	0.50241	0.541343
EPCAT7F524	0.138912	0.139654	0.140402
EPCAT8R190	0.074398	0.071246	0.069917
EPCAT13F999	0.774196	0.782848	0.785502
EPCAT15F850	0.533521	0.534234	0.534993
EPCAT15F849	0.959957	0.972428	0.977337
EPCAT21F823	0.885101	0.864576	0.855339
EPCATXR234	0.091736	0.091129	0.091002
pre-operative PSA	0.000019	0.000022	0.000024
pT-stage	0.081183	0.080368	0.080263
Gleason score	0.000501	0.00026	0.00019

Supplementary Table 5a: Information on clinical follow-up for all four tissue microarrays used for *in situ* hybridization

TMA	TMA1	TMA2 (normal prostate)	TMA3 (LNPCa)	TMA4 (CRPC)
**Clinico-pathological parameter**	Mean (median; IQR) or n (%)	Mean (median; IQR) or n (%)	Mean (median; IQR) or n (%)	Mean (median; IQR) or n (%)
**Age (years)**	64.9 (65.4; 62–68)	66 (66; 62 – 73)	62.5 (65; 61.5–67.8)	70.4 (71; 64.6–75.3)
**PSA level (ng/ml)**				
Total	7.35 (5.2; 3.7–7.8)	-	39.89 (24.7; 11 – 50.8)	67.77 (13.45; 7.25 – 31.25)
≤ 10 ng/ml	366 (88%)	-	22 (19%)	34 (28%)
> 10 ng/ml	52 (12%)	-	74 (62%)	60 (50%)
Unknown	-	-	23 (19%)	26 (22%)
**Follow-up (months)**	116.8 (112; 87.2–136.4)	-	No follow-up registered	No follow-up registered
**Gleason sum**				
< 7	217 (52%)	-	4 (4%)	2 (2%)
7	173 (41%)	-	65 (58%)	7 (6%)
3 + 4	141 (34%)	-	-	-
4 + 3	32 (7%)	-	-	-
> 7	28 (7%)	-	43 (38%)	40 (33%)
Unknown	-	-	-	71 (59%)
**pT-stage (TNM 2002)**				
T2	292 (70%)	-	3 (2,5%)	-
T3a	84 (20%)	-	3 (2,5%)	-
T3b	17 (4%)	-	1 (0,8%)	-
T4	25 (6%)	-	1 (0,8%)	-
Unknown	-	-	111 (93,3%)	-
**Lymph node metastasis**				
Yes	1 (0%)	-	119 (100%)	-
No	417 (100%)	-	-	-
**Surgical margins**				
Positive	110 (26%)	-	-	-
Negative	308 (74%)	-	-	-
**Biochemical recurrence**				
Yes	107 (26%)	-	1 (1%)	-
No	311 (74%)	-	-	-
Unknown	-	-	118 (99%)	-

(*Continued*)

TMA	TMA1	TMA2 (normal prostate)	TMA3 (LNPCa)	TMA4 (CRPC)
**Development of metastasis**				
Yes	20 (5%)	-	41 (35%)	-
No	398 (95%)	-	11 (9%)	-
Unknown	-	-	67 (56%)	-
**Overall death**				
Yes	97 (23%)	-	64 (54%)	51 (42%)
No	320 (77%)	-	51 (43%)	44 (37%)
Unknown	-	-	4 (3%)	25 (21%)
**Death from prostate cancer**				
Yes	11 (3%)	-	44 (39%)	6 (5%)
No	130 (31%)	-	63 (55%)	4 (3%)
Unknown	277 (66%)	-	38 (6%)	110 (92%)
**Hormonal status**				
hormone refractory	-	-	-	65 (54%)
hormone sensitive	-	-	-	55 (46%)
RCP	-	48 (38%)	-	-
TE	-	5 (4%)	-	-
TURP	-	74 (58%)	-	-

RCP – Radical cystoprostatectomy.
TE – Total pelvic exenteration.
TURP – Transurethral resection of the prostate.

Supplementary Table 5b: Information on clinical follow-up for the EMC Exon Array dataset, as well as both qRT-PCR cohorts

	EMC (Exon Array)	EMC discovery (qRT-PCR)	EMC validation (qRT-PCR)
**Clinico-pathological parameter**	Mean (median; IQR) or n (%)	Mean (median; IQR) or n (%)	Mean (median; IQR) or n (%)
**Age (years)**	63.18 (63.5; 58–67)	63.36 (64; 58–67)	68.17 (67; 61–75)
**PSA level (ng/ml)**			
Total	23.37 (10.5; 5.95–19.3)	25.55 (11; 6–20.7)	23.25 (11.25; 5.5–23.85)
≤ 10ng/ml	35 (40%)	29 (38%)	23 (27%)
> 10ng/ml	36 (41%)	32 (43%)	25 (24%)
Unknown	16 (18%)	14 (19%)	46 (49%0
**Follow-up (months)**	124.63 (124; 89–157)	122.56 (126.5; 82.25–159.5)	126.34 (127; 90.5–154.75)
**Gleason sum**			
< 7	36 (41%)	28 (37%)	34 (36%)
7	16 (18%)	13 (17%)	16 (17%)
3 + 4	8 (9%)	7 (9%)	6 (6%)
4 + 3	6 (7%)	4 (5%)	8 (9%)
> 7	23 (27%)	22 (30%)	27 (29%)
Unknown	12 (14%)	12 (16%)	17 (18%)
**pT-stage (TNM 2002)**			
T2	23 (27%)	20 (27%)	15 (16%)
T3a	16 (18%)	14 (19%)	6 (6%)
T3b + c	15 (17%)	11 (14%)	17 (18%)
T4	12 (14%)	10 (13%)	6 (6%)
Unknown	21 (24%)	20 (27%)	50 (53%)
**Lymph node metastasis**			
Yes	12 (14%)	11 (15%)	1 (1%)
No	78 (86%)	64 (85%)	93 (99%)
**Surgical margins**			
Positive	25 (29%)	20 (27%)	17 (18%)
Negative	26 (41%)	20 (27%)	15 (16%)
Unknown	36 (30%)	35 (46%)	62 (66%)
**Biochemical recurrence**			
Yes	45 (52%)	39 (52%)	34 (36%)
No	41 (47%)	35 (47%)	52 (55%)
Unknown	1 (1%)	1 (1%)	8 (9%)
**Development of metastasis**			
Yes	19 (22%)	18 (24%)	19 (20%)
No	67 (77%)	56 (75%)	67 (71%)
Unknown	1 (1%)	1 (1%)	8 (9%)

Supplementary Table 6: Number of tissue microarray samples showing expression (positive) for one EPCAT after *in situ* hybridization. CRPC +: hormone refractory PCa. CRPC −: hormone sensitive PCa

EPCAT	Sample group	TMA1 (PCa)	TMA2 (normal prostate)	TMA3 (LNPCa)	TMA4 (CRPC)
**EPCAT2F176**	total samples	418	127	119	120
	containing tumor	374	0	73	109
	positive (CRPC +/−)	107	0	46	61 (34 / 27)
	Negative (CRPC +/−)	267	127	27	48 (23 / 25)
**EPCAT4R966**	total samples	418	127	119	120
	containing tumor	396	0	71	103
	positive (CRPC +/−)	111	0	16	41 (26 / 15)
	negative (CRPC +/−)	285	127	55	62 (28 / 34)

Supplementary Table 7a: Kaplan-Meier results for two EPCATs based on TMA1 and TMA4 (CRPC). Association with clinical parameters was performed after quantification of *in situ* hybridisation

	PSA at diagnosis (> 10 vs < 10)	Gleason RP	Surg. Margins	pT-stage	CRPC
**EPCAT2F176 (pos/neg)**					
Pearson Chi-Square	0.49	0.368	0.555	0.004	0.417
Likelihood Ratio	0.497	0.372	0.553	0.005	0.417
Linear-by-Linear Association	0.242	0.158	0.555	0.024	0.419
**EPCAT4R966 (pos/neg)**					
Pearson Chi-Square	0.938	0.289	0.346	0.039	0.69
Likelihood Ratio	0.938	0.289	0.0342	0.042	0.68
Linear-by-Linear Association	0.938	0.125	0.346	0.643	0.71

Supplementary Table 7b: Cox regression results for EPCAT4R966 based on TMA1, association with clinical parameters was performed after quantification of *in situ* hybridisation

Cox regression				
**EPCAT4R966 (pos/neg)**	**univariate**		**multivariate**	
**BCR**	**HR (95% CI)**	***p*-value**	**HR (95% CI)**	***p*-value**
Age	1.05 (1.00–1.09)	0.04	1.03 (0.98–1.07)	0.21
PSA	3.51 (2.34–5.26)	< 0.01	1.79 (1.13–2.84)	0.03
Gleason score	2.47 (1.89–3.26)	< 0.01	1.82 (1.34–2.46)	< 0.01
pT-stage	1.72 (1.46–2.02)	< 0.01	1.25 (1.03–1.52)	0.03
Surgical margin	3.29 (2.29–4.71)	< 0.01	2.33 (1.59–3.41)	< 0.01
EPCAT4R966	1.236 (0.819–1.864)	0.319	1.174 (0.775–1.778)	0.448
**local reccurrence**				
Age	0.96 (0.87–1.05)	0.36	0.92 (0.84–1.02)	0.13
PSA	2.28 (0.83–6.22)	0.11	0.96 (0.30–3.10)	0.95
Gleason sum	2.55 (1.35–4.82)	< 0.01	2.08 (1.07–4.05)	0.03
pT-stage	1.60 (1.10–2.35)	0.02	1.21 (0.78–1.87)	0.39
Surgical margin	4.10 (1.73–9.75)	< 0.01	3.27 (1.34–7.95)	0.01
EPCAT4R966	1.684 (0.688–4,122)	0.253	1.763 (0.714–4.353)	0.219
**PCa-death**				
Age	1.10 (1.05–1.15)	< 0.01	1.10 (1.06–1.16)	< 0.01
PSA	1.23 (0.73–2.06)	0.44	1.22 (0.70–2.12)	0.48
Gleason sum	1.29 (0.950–1.760)	0.1	1.21 (0.86–1.70)	0.28
pT-stage	0.98 (0.76–1.22)	0.84	0.88 (0.70–1.12)	0.3
Surgical margin	1.09 (0.72–1.64)	0.68	1.06 (0.70–1.63)	0.8
EPCAT4R966	1.494 (0.970–2.302)	0.069	1.423 (0.923–2.195)	0.111

Supplementary Table 7c: Cox regression results for EPCAT2F176 based on TMA1, association with clinical parameters was performed after quantification of *in situ* hybridisation

Cox regression

**EPCAT2F176 (pos/neg)**	**univariate**		**multivariate**	
**BCR**	**HR (95% CI)**	***p*-value**	**HR (95% CI)**	***p*-value**
Age	1.05 (1.00–1.09)	0.04	1.03 (0.98–1.07)	0.21
PSA	3.51 (2.34–5.26)	< 0.01	1.79 (1.13–2.84)	0.03
Gleason score	2.47 (1.89–3.26)	< 0.01	1.82 (1.34–2.46)	< 0.01
pT-stage	1.72 (1.46–2.02)	< 0.01	1.25 (1.03–1.52)	0.03
Surgical margin	3.29 (2.29–4.71)	< 0.01	2.33 (1.59–3.41)	< 0.01
EPCAT2F176	1.434 (0.961–2.141)	0.078	1.403 (0.932–2.111)	0.105
**local reccurrence**				
Age	0.96 (0.87–1.05)	0.36	0.92 (0.84–1.02)	0.13
PSA	2.28 (0.83–6.22)	0.11	0.96 (0.30–3.10)	0.95
Gleason sum	2.55 (1.35–4.82)	< 0.01	2.08 (1.07–4.05)	0.03
pT-stage	1.60 (1.10–2.35)	0.02	1.21 (0.78–1.87)	0.39
Surgical margin	4.10 (1.73–9.75)	< 0.01	3.27 (1.34–7.95)	0.01
EPCAT2F176	1.396 (0.541–3.601)	0.491	1.605 (0.610–4.219)	0.338
**PCa-death**				
Age	1.10 (1.05–1.15)	< 0.01	1.10 (1.06–1.16)	< 0.01
PSA	1.23 (0.73–2.06)	0.44	1.22 (0.70–2.12)	0.48
Gleason sum	1.29 (0.950–1.760)	0.1	1.21 (0.86–1.70)	0.28
pT-stage	0.98 (0.76–1.22)	0.84	0.88 (0.70–1.12)	0.3
Surgical margin	1.09 (0.72–1.64)	0.68	1.06 (0.70–1.63)	0.8
EPCAT2F176	1.173 (0.762–1.807)	0.468	1.116 (0.721–1.727)	0.624

Supplementary Table 7d: Kaplan-Meier results for two EPCATs and a combination of both based on TMA1, association with clinical parameters was performed after quantification of *in situ* hybridisation

**Kaplan-Meier**	**PSA-progression (BCR)**	**local reccurrence**	**PCa-death**
Log-rank EPCAT2F176	0.075	0.489	0.125
Log-rank EPCAT4R966	0.31	0.248	0.066
Log-rank combination	0.134	0.589	0.223

Supplementary Table 8: Sequences used for RNAscope *in situ* hybridisation on tissue microarrays

EPCAT	Sequence for RNAscope (5′→ 3′)
EPCAT2F176	TGCCATCAATATTCTGAAAATGGCAGTGATTTTTAT<br>TCAACCTGTATAAGGCACTTTCACCATGTACCTGGAAGCAA<br>CATCTACATCTTTTTCAGGTTTTCCTCTGTCCACTATGAAGG<br>ACTTTGTGACCACATTCTGACTCTGATGAGATCCTGCCC<br>AGAATTGACCTGAACCCCAATAATTCACCTTTCTCTCA<br>GGCAATCTAGATGCTGGGGACACAAGGTCCACCTTCCA<br>GGAATATGGCCATGACACCAGAAATCACAAACATGATG<br>AGAATGGAATGACTGGGGAAGAAGTGCCAGATGCTTCA<br>CTTGTAAATGAAGACCCAGCCTCTGGGGATGCAGATA<br>CCACCTCCCTGAAGAAGCTGAATATCTGCAGATAAGTG<br>GAGTTCACCAATGATGAGGAGCGGGATGGAGAAAGGA<br>GGTAGGGAGAGTCATCCAAGGAACATGA<br>GCAACATGTTAAAAGG
EPCAT4R966	GTGACACAGAGAATGAGTGGAATACTAATACATT<br>GAAGGGCAAAATCAAAACCCCAGAA<br>GATCTCTAAAAGTCTGAATGAATCAGCCCATATTACA<br>AGATAAAGTCCATAATGGTAAACGGAAGGCTCTGG<br>TCTTGGTTTACAAAATCCAACCACTCAAATATGTAG<br>TTGCTGGAAGTTTTGTGGCCATGGGGATGCTGACAAA<br>GATTGCTTGGGATGCACACAAGGCAGCGGCGAAGCCT<br>GAGGGGTGCTGCCAGGCCGGGCTCTCCACTGGCCACG<br>AATTTGTACATATGGCTTTGGAAACCTCATCCAACAC<br>TAAGTAATGAACATACATGAGGATACTATGTATTTTTCAACC<br>TGCAAGCCCAAGATTTCAAATTCTTTGGTTTATCGCA<br>GAGAACACATCTCTCAAATTGTCTTCAAGGAGC<br>ACTGCAGGAGAAGTCCTGGCAACTTGGCGCAGCGAAT<br>TTGATCAGCACCATACGTACGACCTGTCTCAGGAACG<br>GTGCCATGTGTGTGACTAGGACTGCAAAGCAGCTT<br>GTGTTCGTGGGGGAAATGACAGGCACAGGAAGGAAA<br>AGCTGAGAACACTGAAACGGTTCCTAATCGTGCAGTGG<br>GGAAAGGAGGGCGACTGCCGCGCAGAGTGGCACGTT<br>GGCTGGAAGAGGGGATTGATCAGCAGTGCTGGCAGGAA<br>CAGCATAGGCTTGTCGTGTGATCTAACAGAAGAGGC<br>AGCAATCTGGAGGCGCCCCAGATCAGAGAAGAGACATCA<br>AGCCCCAGGAGAAATGGATTTCCTTTCTCCCCACC<br>CATCAGCTGGGTAGGAAGCCCGAAGGAGCCTTTCATGGAAC<br>TATGGGAAGGGCTCCTCTGACGCAGAGGGGATG<br>AACTGGAAGCAAGATGCTGACTGTTTCCAAAG<br>GCTTGACAGTCAGAAG<br>CAGGCAGGCTTCAGCCTATTGAGATACTTTACCC<br>AAGAGGATTTCCGCTTAGGTTCT<br>GTCTTGAAAGAAATATTCGCTCCCA<br>AATTTCCTCCCATCAAGAGTGCTTCT<br>CCCTACAGAAGGCTAAGGCTCACAGGTTGTCCCCAGGGA<br>CACATTTCTTTCAAGTAG<br>GATGACTGGTATGTTTAGACTGAAAAGCTGA<br>TTTACTGGAAGATTGCTATCACCC<br>CCTGGCCTACCATGTC

Supplementary Table 9: Sequences of working Taqman probes used for quantitative real-time PCR quantification of 11 EPCATs

name	target exon junction	forward primer sequence	reverse primer sequence	sequence 5' FAM 3' TAMRA probe
EPCAT1F273	J–K	CAGAGCTTTCACTCTAGCATCTGA	CAGTTGTAGGCATCGGTGAA	TTATCATGGGCCTGGATCTT
EPCAT2F176	A–B	GAACCCACCAGAAGGAAAAA	GCTTCCAGGTACATGGTGAAA	TGTAACACTCACTGCGAGGG
EPCAT2R709	A–D	CAGTGCATGGACATTTGAGC	GGCATGACAGAGGAAACAATTC	TGGAGCATAACCAACCTT CAGAACTTGT
EPCAT3R522	B–C	GTGTTAGTGCATTGGAAAATCA	TTTCTCTCTCAGTCCAGCAATG	TCCTGCCAGGCTTTGACTAC
EPCAT4R966	B–C	TGCTGACAAAGATTGCTTGG	TGCAGTGCTCCTTGAAGACA	GCTTTGGAAACCTCATCCAA
EPCAT5F602	B–C	GGCAATGGAATGACCATTTT	ATGGTTCCTCTCACGACTGG	ACCTATGGGCCATAGCCTTT
EPCAT5R633	A–B	TGATCAGTGGCAGAAAGAGC	ACGAGGTGAGAAATCCGTCA	AGGGAAAAGGAGCACAGGAT
EPCAT7F524	A–B	TTTGCATGGTGTTTTGGTGT	CCGATGTCTGTCATCTGGTG	GGAGAGAGGATGGCATACCA
EPCAT8R190	D–E	AGAGCCACCTTAGCCAATGA	AAGACCCCCAAATCTCATCC	AGTTCAGGAAGCAGGCACAT
EPCAT15F850	A–B	GGATGCACCGTCACCTATG	GCCACAACTTCCATAACTTCC	CCGGCACGCAAGAT GTGTATTCTCA
EPCATXR234	C–D	ACACTGTTCATCCCCACTCC	TTTTGTTCCCGTC CTTCTTG	TTTGCACACAGAAAAGCACC

Supplementary Table 10: Primer sequences used for validating exons/exon pairs (junction spanning). Primers were considered working (Y) if a band of the expected size was present after electrophoresis. Primers were considered not working (N) if the expected band was missing, differed from expected size or the reaction mix without reverse transcriptase also contained a band of the expected size
